# Origin site‐based staging system of sinonasal inverted papilloma for application to endoscopic sinus surgery

**DOI:** 10.1002/hed.25435

**Published:** 2018-12-15

**Authors:** Yifan Meng, Gaoli Fang, Xiangdong Wang, Xiaohong Song, Kuiji Wang, Hongfei Lou, Wenyu She, Long Qin, Mei Lv, Tao Zhang, Tiejun Yuan, Yanli Tao, Xun Meng, Luo Zhang, Chengshuo Wang

**Affiliations:** ^1^ Department of Otolaryngology, Head and Neck Surgery Beijing TongRen Hospital, Capital Medical University Beijing China; ^2^ Department of Otolaryngology, Head and Neck Surgery Beijing Ditan Hospital, Capital Medical University Beijing China; ^3^ Department of Allergy Beijing TongRen Hospital, Capital Medical University Beijing China; ^4^ Department of Otolaryngology, Head and Neck Surgery The Second Affiliated Hospital of Baotou Capital Medical University Inner Mongolia China; ^5^ Department of Otolaryngology, Head and Neck Surgery The First Affiliated Hospital of Dalian Medical University Liaoning China; ^6^ Department of Otolaryngology, Head and Neck Surgery The First Affiliated Hospital of JiNan University Guangdong China; ^7^ Department of Otolaryngology, Head and Neck Surgery Weifang People's Hospital Shandong China; ^8^ Key Laboratory of Otolaryngology, Head and Neck Surgery Ministry of Education, Beijing Institute of Otolaryngology Beijing China

**Keywords:** endoscopy, recurrence, sinonasal inverted papilloma, staging system, surgical approach

## Abstract

**Background:**

We aimed to assess the recurrence risk of sinonasal inverted papillomas (SNIPs), based on a staging system developed according to the originating site of SNIP.

**Methods:**

A total of 200 patients with SNIP were enrolled, and a staging system was developed based on the originating sites and corresponding recurrence rates of tumor in the patients. In the verification phase, 675 patients with SNIPs were enrolled as above, and the originating sites of the SNIPs were confirmed by an endoscopic sinus surgery. Cluster analysis was performed to determine the stage for each SNIP.

**Results:**

Overall, 608 patients completed the study. SNIP recurrence rates for stages 1‐4 were 0 (n = 43), 4.0% (n = 420), 13.4% (n = 134), 36.4% (n = 11), respectively (total = 6.4%).

**Conclusions:**

The origin site‐based classification of SNIP may aid surgeons in selecting appropriate endoscopic surgical approaches to minimize the risk of recurrence.

## 1 INTRODUCTION

Sinonasal inverted papilloma (SNIP) is a type of benign tumor located in the sinonasal area.^1^ It arises in the Schneiderian epithelium and represents 0.5%‐4% of all sinonasal tumors.^2^, ^3^ A SNIP, however, has the potential for malignant transformation, with some studies reporting a malignancy rate of about 10%.^4^, ^5^ Despite the malignancy, a most challenge aspect of SNIP for otolaryngologists is the high recurrence rate of this entity.^2^ Indeed, several studies have indicated that the recurrence rate of SNIP may range from 10% to 25.3%.^6^, ^7^, ^8^ Although endoscopic surgery is the gold standard for the treatment of SNIP, the major factor associated with recurrence appears to be the incomplete initial resection of the SNIP,^2^, ^9^ particularly as most SNIP recur at the same site from which the tumor originated.^5^, ^10^ Thus, identification of the origin site of a SNIP before surgery and complete resection of the original location and surrounding area of the tumor are important for preventing or reducing the risk of recurrence after surgical intervention.^10^

The identification of the origin site of SNIP is essential for the management of this tumor, which sometimes has a small narrow pedicle or attachment.^11^ With the development of endoscopic sinus surgery (ESS), CT, and MRI, it is now possible to trace the original site of the tumor, regardless of the tumor size.^11^ Although Chawla et al.^12^ have demonstrated that the osteitis sign and neo‐osteogenesis on the CT scans could be used to identify the site of attachment of the SNIP, Fang et al.^10^ have indicated that preoperative MRI may provide a better option to accurately predict the original site of the SNIP than CT, and thus may facilitate accurate and complete removal of the SNIP.^10^

Staging is important in prognosis of the disease^9^ and in achieving a satisfactory surgical outcome.^11^ In this regard, several different SNIP staging systems have been reported and commonly used over the last few decades.^13^, ^14^, ^15^, ^16^ Almost all the published staging systems to date have been based on tumor volume,^1^ and based on this logic, a larger tumor has often meant higher staging and higher recurrence risk. However, Kim et al.^11^ have demonstrated that there was no significant difference between SNIP recurrence rate and clinical stage based on different staging systems, including the Krouse staging system,^13^ Furuta staging system,^17^ and Citardi staging system.^16^ Although some studies have recommended staging the SNIP based on the original site of the tumor rather than the tumor volume,^11^, ^18^, ^19^ none of the SNIP staging systems reported in recent years were shown to accurately associate recurrence rates according to SNIP stages.^13^, ^14^, ^15^, ^16^ The aim of this study was to assess the recurrence risk associated with different SNIP origin sites and establish an ESS staging system based on the SNIP origin site.

## 2 PATIENTS AND METHODS

### 2.1 Patients and study design

Patients diagnosed with SNIP based on MRI findings, as we have described earlier,^10^ and histopathological evaluation of biopsy samples were enrolled from 6 centers across China. All centers used the same diagnostic and preoperative procedures for the assessment of eligible patients with SNIP, and patients with tumors demonstrating malignant transformation were excluded because the therapeutic strategy would be altered accordingly. Before surgery, the demographic characteristics of each patient and the origin and affected sites with SNIP were recorded. The surgical approaches and the exact origin and involved sites of the tumor were recorded, and all the patients were followed up for at least 36 months, during which period SNIP recurrence times and recurrence rates were recorded.

This study was approved by the ethics committees of the 6 participating centers: Beijing TongRen Hospital, Beijing Ditan Hospital, The Second Affiliated Hospital of Baotou Capital Medical University, The First Affiliated Hospital of Dalian Medical University, The First Affiliated Hospital of JiNan University, and Weifang People's Hospital; and conducted in accordance with the ethical standards of the committee on human experimentation of the institution or in accordance with the Helsinki Declaration of 1975 as revised in 1983. All patients were given the relevant study information and instructions necessary for inclusion in the study, and all patients provided written informed consent before entering into the study.

### 2.2 Staging system

#### 2.2.1 Developmental phase

**Figure 1:**
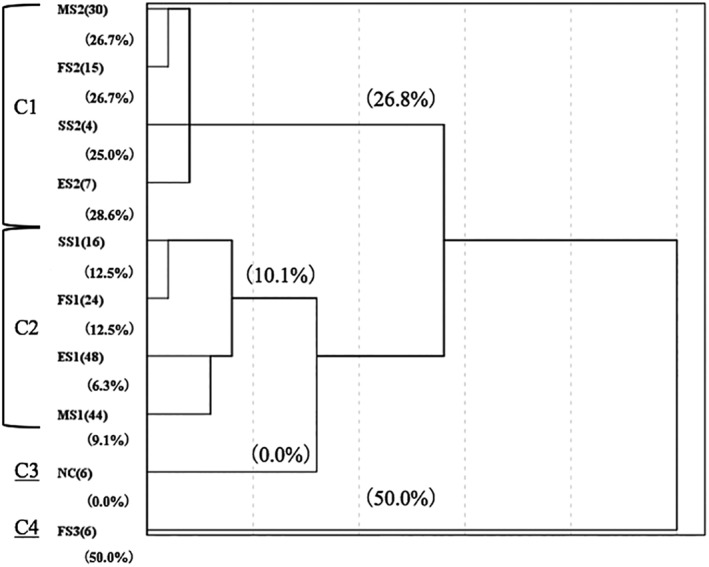
Cluster analysis of data for 200 patients enrolled in the preliminary study from March 2000 to March 2007. Four clusters were determined based on recurrence rates for different anatomical regions. NC, nasal cavity; ES1, ethmoid sinus (except superior orbital cell); MS1, posterior, lateral, or superior wall of the maxillary sinus; SS1, sphenoid sinus (medial of Sternberg's canal); FS1, frontal sinus (facial midline to lamina papyracea); ES2, superior orbital cell; MS2, inferior, anterior, or medial wall of the maxillary sinus; SS2, sphenoid sinus (lateral of Sternberg's canal or affected bilateral sinus); FS2, frontal sinus (lamina papyracea to central pupil or invading bilateral sinus); FS3, frontal sinus (lateral of central pupil)

Preliminary investigations were conducted in 200 patients with SNIP, who underwent ESS in the 6 designated study centers from March 2000 to March 2007. The originating and involved sites of SNIP predicted by MRI evaluation^10^ were confirmed by the surgical procedure, and the patients were followed up for a minimum of 36 months, during which period the patients were evaluated for recurrence of the SNIP. Retrospective analysis of data obtained from this cohort indicated that the SNIP recurrence rates were different according to the site from which the tumor originated. Thus, when the tumor originated from a location that was easy to access—for example, the nasal cavity, ethmoid sinus, posterior‐lateral and superior wall of the maxillary sinus—the rates of recurrence were low (less than 10%). However, when the tumor originated from a location that was more difficult to access—such as lateral partial of frontal sinus—the recurrence rate was significantly increased (50% in this cohort). In view of this finding, we summarized 10 anatomic regions according to different originating site and difficulty of accessing and then performed cluster analysis based on the recurrence rate. This analysis indicated 4 clusters (Figure 1), including cluster 1: ethmoid sinus (except supraorbital ethmoid cell), posterolateral or superior wall of the maxillary sinus, sphenoid sinus (except lateral recess of sphenoid sinus), frontal sinus (facial midline to lamina papyracea); cluster 2: superior orbital cell, inferior, anterior, or medial wall of the maxillary sinus, lateral recess of sphenoid sinus or invading bilateral sphenoid sinus), frontal sinus (lamina papyracea to central pupil or invading bilateral frontal sinus); cluster 3: nasal cavity; and cluster 4: frontal sinus (lateral of central pupil).

**Figure 2:**
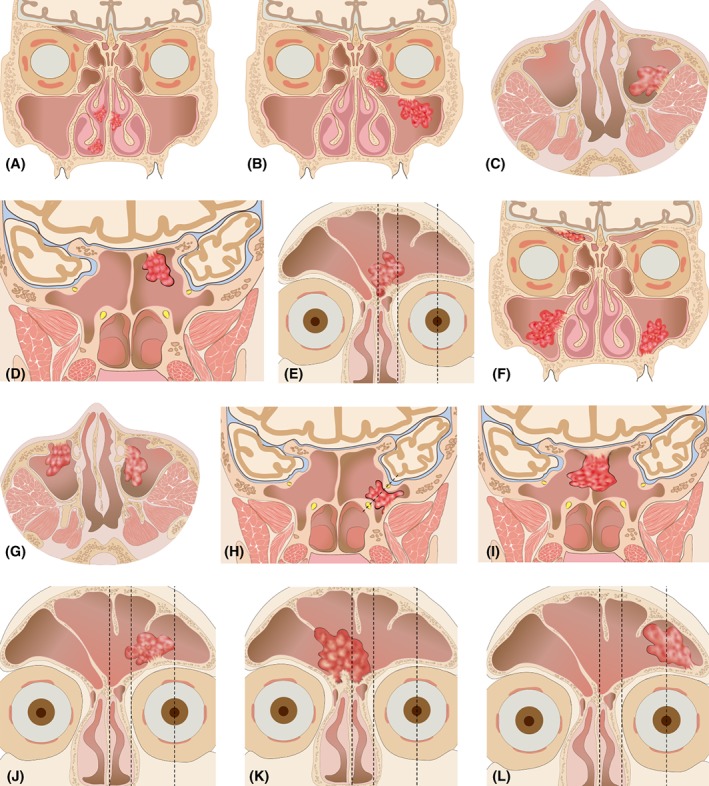
Schematic diagram showing the endoscopic staging system of sinonasal inverted papilloma (SNIP), based on the tumor origin sites. (A) Stage 1: origin site located in nasal cavity; (B) stage 2: origin site located in ethmoid sinus (except superior orbital cell) and superior wall of the maxillary sinus; (C) stage 2: origin site located in lateral, posterior wall of the maxillary sinus; (D) stage 2: origin site located in sphenoid sinus (medial of Sternberg's canal); (E) stage 2: origin site located in frontal sinus (facial midline to lamina papyracea); (F) stage 3: origin site located in the superior orbital cell or inferior wall of the maxillary sinus; (G) stage 3: origin site located in anterior or medial wall of the maxillary sinus; (H) stage 3: origin site located in sphenoid sinus (lateral of Sternberg's canal); (I) stage 3: origin site located in sphenoid sinus septum and bilateral sinus; (J) stage 3: origin site located in frontal sinus (lamina papyracea to central pupil); (K) stage 3: origin site located in bilateral fontal sinus and break through the frontal sinus septum; (L) stage 4:origin site located in frontal sinus (lateral of central pupil)

Based on these clusters, we further categorized a SNIP into 4 stages as follows: stage 1 (cluster 3): the region that was easiest to access with an endoscope and the surgeon could resect the tumor directly without further sinusotomy; stage 2 (cluster 2): anatomic regions that were easy to access and sinusotomy was necessary for resecting the tumor origin site; stage 3 (cluster 1): anatomic regions that were difficult to access even after sinusotomy, and the surgeon needed to adopt a specialized endoscopic approach; stage 4 (cluster 4): anatomic regions that were scarcely possible to access using an endoscopic approach (such as far corner of frontal sinus), and therefore ESS in combination with an external approach was recommended (Table 1 and Figure 2).

**Table 1:** The sinonasal inverted papilloma staging system and recommended surgical approach

Classification based on the originating site		Recommended surgical approach
Stage 1	Nasal cavity	ESS (simple tumor resection)
Stage 2	Ethmoid sinus (except superior orbital cell)	ESS
	Posterior, lateral, or superior wall of the maxillary sinus; sphenoid sinus (medial of Sternberg's canal); frontal sinus (facial midline to lamina papyracea)	
Stage 3	Superior orbital cell	ESS‐assisted endonasal approach (ie, Draf IIb approach plus lamina papyracea resection)
	Inferior, anterior, or medial wall of the maxillary sinus; sphenoid sinus (lateral of Sternberg's canal)	ESS‐assisted prelacrimal duct approach
		ESS‐assisted pterygoid process approach
	Sphenoid sinus (invading bilateral sinus)	ESS‐assisted sphenoidal rostrum process approach
	Frontal sinus (lamina papyracea to central pupil or invading bilateral sinus)	Draf IIb approach plus lamina papyracea resection or ESS‐assisted Draf III approach
Stage 4	Frontal sinus (lateral of central pupil)	ESS with combined external approach

Abbreviation: ESS, endoscopic sinus surgery.

#### 2.2.2 Verification phase

To verify this staging system, we performed a prospective multicenter study involving 675 patients with SNIP enrolled from the same 6 designated hospitals in China, from March 2010 to February 2015. The originating sites of SNIP were evaluated by surgery, using standardized experimental and surgical approaches in all the centers, and staged accordingly (Table 1). In particular, for the SNIPs with originating site included in stage 1 or stage 2, the surgeons used standard ESS to excise the tumor. Similarly, in stage 3, for tumors originating from superior orbital cell, the ESS‐assisted Draf IIb approach plus lamina papyracea resection was used; for tumors originating from inferior, anterior, or medial wall of the maxillary sinus, the ESS‐assisted prelacrimal duct approach was used; for tumors originating from sphenoid sinus (lateral of Sternberg line), the ESS‐assisted pterygoid process approach was used; for tumors originating from bilateral sphenoid sinus (break through sphenoid sinus septum), the ESS‐assisted sphenoidal rostrum process approach was used; and for tumors originating from frontal sinus (lamina papyracea to central pupil or invading bilateral frontal sinus), the ESS‐assisted Draf IIb approach plus lamina papyracea resection or Draf III approach was used; whereas in stage 4, for tumors originating from frontal sinus (lateral of central pupil), the ESS with a combined external approach, such as the eyebrow incision, was used (Table 1).

If the tumor originated from more than 1 site, then it was categorized into the highest stage based on the originating sites.

### 2.3 Statistical analysis

Hierarchical clustering method was performed using SPSS 22.0 (SPSS Inc., Chicago, Illinois), the different origin site of tumor and corresponding recurrence rate were enrolled in the statistical model, and the result was rendered as a cladogram. Chi‐square or Fisher's exact test was used to analyze recurrences rates, and the results were considered statistically significant at *P* < .05. GraphPad Prism 6.0 software (GraphPad Software, Inc., La Jolla, California) was used for survival analyses. Survival was calculated using the Kaplan‐Meier method and curves compared using Mantel‐Haenszel test (log rank). End points were calculated from the date of diagnosis until recurrence.

## 3 RESULTS

A total of 608 of 675 patients (531 men, 77 women; mean age 49.9 ± 4.5 years) completed the study, with a mean follow‐up time of 60.3 ± 2.7 months. The total recurrence rate was 6.4%, with recurrence rates of 0, 4.0%, 13.4%, and 36.4% for stages, 1, 2, 3, and 4 tumors, respectively (Table 2).

**Table 2:** The characteristics of different stage

	Stage 1	Stage 2	Stage 3	Stage 4	*P* value
Case	43	420	134	11	
Age (mean ± SD), years	45.7 ± 7.4	50.7 ± 7.1	50.0 ± 4.3	53.1 ± 3.8	NS
Sex (M/W)	29/14	381/39	113/21	8/3	<.001
Recurrence cases (%)	0 (0)	17 (4.0)	18 (13.4)	4 (36.4)	<.01

Abbreviations: M, men; W, women.

Based on the current staging system, in stage 1, the tumor originated from the nasal cavity in 43 cases, whereas in stage 2, the tumor originated from maxillary sinus in 97 cases (23.1%), from ethmoid sinus in 255 cases (60.7%), from frontal sinus in 38 cases (9.0%), and from sphenoid sinus in 30 cases (7.1%) (Table 2). Similarly, in stage 3, the tumor originated from maxillary sinus in 72 cases (53.7%), from frontal sinus in 29 cases (21.6%), from sphenoid sinus in 19 cases (14.2%), and from superior orbital cell in 14 cases (10.4%), whereas 11 cases of tumor originating from lateral partial of frontal sinus were noted in stage 4 (Table 2). Overall, ethmoid sinus (44.2%) and maxillary sinus (27.8%) were most affected, followed by frontal sinus (12.8%) and sphenoid sinus (8.1%), according to our staging system (Table 3). Assessment according to the Krouse staging system^13^ also demonstrated that the ethmoid sinus (41.8%) and maxillary sinus (29.1%) were also most involved, followed by frontal sinus (11.7%) and sphenoid sinus (10.7%) (Table 3).

**Table 3:** SNIP originating site located in sinuses based on the staging system employed

Classification based on:		
SNIP originating site (%)		Krouse Classification^13^ (%)
Stage 1	43 cases	23 cases
Stage 2	420 cases	
	97 in maxillary sinus (23.1)	322 cases
	255 in ethmoid sinus (60.7)	68 in maxillary sinus (21.1)
	38 in frontal sinus (9.0)	254 in ethmoid sinus (78.9)
	30 in sphenoid sinus (7.1)	
Stage 3	134 cases	
	72 in maxillary sinus (53.7)	245 cases
	29 in frontal sinus (21.6)	109 in maxillary sinus (44.5)
	19 in sphenoid sinus (14.2)	71 in frontal sinus (29.0)
	14 in superior orbital cell (10.4)	65 in sphenoid sinus (26.5)
Stage 4	11 cases	18 cases
	11 in frontal sinus	

**Figure 3:**
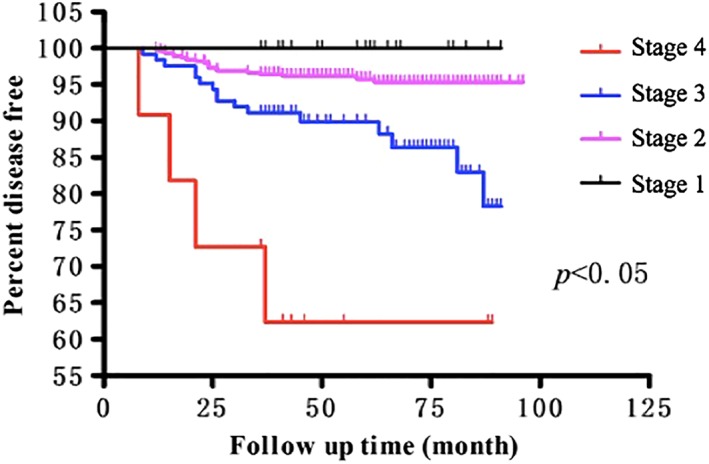
Survival analysis of the study cohort. The recurrence‐free survival rates of stages 1‐4 were 100% (black line), 96.0% (92.4%‐98.1%, pink line), 86.6% (83.1%‐94.7%, blue line), and 63.6% (56.7%‐70.7%, red line), respectively (95% confidence interval, *P* < .05)

The survival analysis for the study cohort is shown in Figure 3. The estimated recurrence‐free survival rates (mean [95% confidence interval]) from stages 1, 2, 3, and 4 after surgery were 100%, 96.0% (92.4%‐98.1%), 86.6% (83.1%‐94.7%), and 63.6% (56.7%‐70.7%), respectively. Representative MRI/CT scans and nasal endoscopic examination images of SNIP origin in individual patients at different stages, before and after surgery using different surgical approaches based on the staging of the tumors, are shown in Supporting Information Suppl. Figures 1‐6.

## 4 DISCUSSION

SNIP is a common tumor, with a high recurrence rate ranging from 10% to 25.3%.^6^, ^7^, ^8^ It has been suggested that the risk of recurrence of SNIP is likely to be a consequence of incomplete surgical resection, rather than due to specific characteristics of the tumor itself,^20^, ^21^ particularly because most SNIP recur at the same site where the tumor originated.^5^, ^10^ Thus, the concept of SNIP resection has changed from aggressive surgery to pedicle‐oriented endoscopic surgery.^2^, ^5^, ^6^, ^22^ However, some studies have recommended routine endoscopic surgery combined with an advanced endonasal approach or external approach, because some sites of the tumor origin were located in anatomic areas, which were hard to access by endoscopy alone.^23^, ^24^

In this study, we performed a retrospective cluster analysis of data from a preliminarily investigation involving a cohort of 200 patients with SNIPs from 6 centers and developed a staging system for assessment of SNIP recurrence risk and appropriate surgical approach recommended, based on the originating site of the tumor. Treatment of patients using surgical approaches recommended according to this staging system in a subsequent prospective multicenter study involving 675 SNIP patients demonstrated a total recurrence rate of 6.4% for this cohort, which was much lower compared with several earlier large sample studies.^6^, ^7^, ^8^ Moreover, our study demonstrated that the recurrence rate increased and conversely the estimated recurrence‐free survival rate decreased, with increasing order of SNIP staging. However, in accordance with findings from other studies,^5^, ^9^ our study also demonstrated that SNIP originated mostly in ethmoid sinus (44.2%), followed by maxillary sinus (27.8%), frontal sinus (12.8%), and sphenoid sinus (8.1%). Furthermore, these results were comparable with those noted using the Krouse staging system.^13^

In the case of the 43 patients in stage 1, none had recurrence, likely because the tumor originating in the nasal cavity (nasal septum or inferior turbinate) could be observed readily using 0° endoscopy and the tumor pedicle resected completely (Supporting Information Suppl. Figure 1). In the case of 255 patients in stage 2 with tumors originating in the ethmoid sinus (except superior orbital cell); the tumors in 184 of these patients originated from the anterior ethmoid sinus and in the remaining patients from the posterior ethmoid sinus. However, regardless of location of the tumor origin in the ethmoid sinus, the total recurrence rate was low, possibly because, similar to stage 1 tumor, the tumor origin site and pedicle of SNIP could easily be visualized^5^ using 0° endoscopy or angle endoscopy, and sinusotomy and complete resection of the tumor pedicle achieved successfully. Furthermore, the tumor origin sites in the nasal cavity or ethmoid sinus (except superior orbital cell) were easy to follow up after surgical intervention for signs and symptoms of recurrence, such as nasal obstruction and epistaxis, and administration of appropriate timely treatment.^5^, ^11^ In this regard, tumors originating from superior orbital cell were an exception. Thus, in the case of 14 patients with tumors originating from the superior orbital cell in this study, there was recurrence in 2 of these patients; which led to significantly greater recurrence rate than for tumors originating from the ethmoid sinus (*P* < .05). However, as the tumor originating site was sometimes located at the most distal part of the superior orbital cell, partial resection of the lamina papyracea and displacement of the eye ball to the outside to identify the originating site of the SINP for resection were necessitated. Thus, although the superior orbital cell belongs to anterior ethmoid sinus, in this study a SNIP in this area was categorized as stage 3.

Similarly, there were 169 patients with SNIPs originating from the maxillary sinus, with tumors in 97 patients originating from lateral, posterior, or superior wall of the maxillary sinus and tumors in the remaining 72 patients originating from inferior, anterior, or medial wall of the maxillary sinus. In the case of patients with tumors originating from lateral, posterior, or superior wall of the maxillary sinus, these were categorized as stage 2, because tumor resection could be achieved more easily through maxillary sinus ostium using 0° endoscopy or angle endoscopy, and with a reduced recurrence rate (Supporting Information Suppl. Figure 2). However, in the case of patients with the tumors originating from inferior, anterior, or medial wall of the maxillary sinus, these were categorized as stage 3, because it was difficult to observe the tumor's originating site due to the acute angle, even using 70° endoscopy. Indeed, the recurrence rate was significantly higher in these groups of stage 3 patients compared with their counterpart stage 2 patients (11.1% vs 3.1%; *P* < .05). Thus, in view of this finding, we recommended that for patients with tumor originating from inferior, anterior, or medial wall of the maxillary sinus, ESS‐assisted prelacrimal duct approach should be used to manage the SNIP more directly and easily (Supporting Information Suppl. Figure 3), as demonstrated by Suzuki and colleagues.^23^ Indeed, several studies have suggested that ESS‐assisted prelacrimal duct approach was a safe and effective method for resecting SNIP from maxillary sinus with low recurrence rate.^11^, ^23^, ^24^ Furthermore, the use of this technique may avoid the sacrifice of the inferior turbinate and other intranasal anatomic structures, thereby reducing the possibility of further abnormal physiological effects.^24^

The management of SNIP originating from the frontal sinus is the most challenging due to the narrow space, the anatomically variable frontal recess, and the proximity to the orbital tissue and anterior skull base.^25^ In this study, we divided the frontal sinuses into 3 zones according to facial midline, lamina papyracea, and central pupil (Figure 2E,J,K,L). When the SNIP originated from facial midline to lamina papyracea, sinusotomy and tumor resection were relatively easier, and this was categorized as stage 2. However, when the tumor originated from lamina papyracea to central pupil or invaded bilateral frontal sinus, the originating sites in these areas were difficult to observe due to the acute angle,^26^ and these were categorized as stage 3. For these types of SNIP, we recommend the use of Draf IIb plus lamina papyracea resection approach or ESS‐assisted Draf III approach (Supporting Information Suppl. Figure 5). Zhang et al.^21^ and Takahashi et al.^25^ have suggested that the Draf III surgical approach allows good visualization of a tumor's originating site and should therefore be used to treat SNIP because it can avoid facial destruction.

In this study, 11 patients were seen with SNIPs located in lateral of central pupil, which required an ESS‐combined external approach for excision, and were therefore categorized as stage 4. The overall tumor recurrence rate for this stage was significantly higher compared to the other stages; with 4 of these patients (36.4%) experiencing recurrence. The SNIP patients in this stage always demonstrated either one or both very well‐pneumatized frontal sinuses, which were beyond the same side vertical line of central pupil. For SNIP originating site located in this area, it was rarely possible to achieve complete endonasal pedicle resection using even the Draf III approach, because of the difficulty in accessing the site with the forceps or microdebrider; thus in such cases, we recommend the use of ESS with a combined external approach (Supporting Information Suppl. Figure 6). This finding is in accordance with the findings of Timperley et al.,^27^ who assessed endoscopic access to the frontal sinus in 10 cadaver heads. The authors divided the unilateral frontal sinus into 4 zones, of which zone 1 comprised the medial quarter, zone 2 from zone 1 to the midorbital point, zone 3 from zone 2 to halfway between the midorbital point and the lateral aspect of the orbit, and zone 4 comprised the lateral‐most quarter. In this regard, zones 1 and 2 were comparable to stage 3, and zones 3 and 4 were comparable to stage 4 in this study. The authors demonstrated that although the frontal sinus zones 1 and 2 could be accessed completely using the Draf III approach, only 10% of the orbital roofs in the lateral partial of frontal sinus (zones 3 and 4) were accessible using this approach and ancillary external approaches may be required for surgery in these areas.^27^Similarly, other studies have also suggested that when the tumor was located in the lateral of frontal sinus, ESS with combined external approach was indicated and had lower tumor recurrence rate. 17, 28, 29

In accordance with other studies,^5^, ^9^ this study has also demonstrated that the sphenoid sinus is the least affected, with only 49 patients (8.1%) demonstrating a SNIP in this area. We found that some patients had well‐pneumatized sphenoid sinus lateral recess and therefore required different endonasal approaches according to the different locations of tumor originating sites in the sphenoid. Thus, the unilateral sphenoid sinus was divided into medial part and lateral recess of the sphenoid sinus, and similar to frontal sinus, when the SNIP originated close to the medial part of sphenoid sinus (stage 2), tumor resection was recommended using 0° endoscopy or angle endoscopy. However, when the tumor originated in lateral recess of the sphenoid sinus (stage 3). ESS‐assisted pterygoid process approach was recommended due to the challenges posed by the presence of closely situated vital structures such as the optic nerve and the carotid artery.^30^ Although one study has suggested that endonasal pterygoid process approach could take advantage of the vidian nerve to locate the position of foramen lacerum of the internal carotid artery,^31^ another study has set up a classification to help to understand anatomy of pterygoid area to achieve endoscopic endonasal transpterygoid approach.^32^ In this study, 9 tumors were found to break through the sphenoid sinus septum and invade bilateral sphenoid sinus (stage 3, Supporting Information Suppl. Figure 4). Thus, ESS‐assisted sphenoidal rostrum process approach was performed to resect the sphenoid sinus septum and then integrate bilateral sphenoid sinuses.

## 5 CONCLUSION

Incomplete surgical resection of the originating site of a SNIP is the most important risk factor of recurrence and thus the development of a SNIP staging system associated with recurrence rate based on the originating site of tumor was the main focus of this study. The origin site‐based classification of SNIP we have developed is easy to understand and demonstrates a good correlation between the SNIP stage and recurrence rate. It is possible that identification of the originating site of a SNIP before or during a surgical intervention may aid surgeons to select appropriate endoscopic surgical approaches recommended according to the staging system, thereby minimizing the risk of recurrence.

## CONFLICT OF INTEREST

The authors declare that they have no financial or personal relationships with persons or organizations that can inappropriately influence their work. Furthermore, there is no professional or other personal interest of any nature or kind in any product, service and/or company that could be construed as influencing the position presented in, or the review of this manuscript.

## Supporting information


**Figure S1** Coronal contrast‐enhanced T1‐weighted MRI image (a) and nasal endoscopic examination (b) of a 45 years old woman, showing sinonasal inverted papilloma originating from left inferior turbinate (a, black circle). Endoscopic sinus surgery (ESS) was performed to remove the tumor, and 6 years after left inferior turbinate resection, there is no recurrence to date, as indicated by absence of tumor by CT scan (c) and endoscopy (d). **Figure S2** Axial CT scan (a) and contrast‐enhanced T1‐weighted MRI image (b) of a 55 years old male, showing sinonasal inverted papilloma originating from posterior wall of left maxillary sinus (a, black circle; b, white circle). Endoscopic sinus surgery (ESS) was performed to remove the originating site of the tumor under 70° endoscopy (c ‐ tumor indicated by black arrow; d ‐ the tumor origin site after burning); with no recurrence to date 3 years after endoscopic sinus surgery. **Figure S3** Axial (a) and coronal (b) contrast‐enhanced T1‐weighted MRI image of a 49 years old male, showing sinonasal inverted papilloma originating from anterior wall of left maxillary sinus (white circles). Endoscopic sinus surgery (ESS)‐assisted prelacrimal duct approach surgery was performed to remove the tumor (c ‐ black arrow indicates the originating site of the tumor, the white dotted lines indicated the lacrimal duct). Suppl. Figure 3d shows the origin site under 70° endoscopy after tumor resection (white dotted circle), A, I, M represented anterior, inferior, and medial wall of the maxillary sinus respectively; with no tumor recurrence after 5 years' follow‐up. **Figure S4** Coronal CT scan (a) and contrast‐enhanced T1‐weighted MRI image (b) of a 59 years old female, showing sinonasal inverted papilloma originating from sphenoid sinus septum and affecting bilateral sinus. Endoscopic sinus surgery (ESS)‐assisted sphenoidal rostrum process approach surgery was performed to remove the tumor (c shows the endoscopic image after sphenoid sinus septum resection [white dotted line]; and d shows the CT scan 3 years after surgery, with no tumor recurrence to date). **Figure S5** Coronal (a) and axial CT (b) scans and contrast‐enhanced T1‐weighted MRI images (c, d) of a 46 years old woman, in whom the sinonasal inverted papilloma originated from bilateral frontal sinus (e, black arrows and * indicate SNIP in the left and right frontal sinus, respectively). Endoscopic sinus surgery (ESS)‐assisted Draf III approach surgery was performed to remove the tumor (f ‐ endoscopic image after ESS). Suppl. Figure 5 g and 5 hours show the CT scans at 10 days after surgery and Suppl. Figure 5i the endoscopic image of the patient 5 years after surgery, with no tumor recurrence to date. **Figure S6** Coronal contrast‐enhanced T1‐weighted MRI image (a) of a 61 years old man, showing sinonasal inverted papilloma origin from lateral partial of right frontal sinus (white arrow). Despite employing a ESS‐Draf III surgical procedure and partial resection of left lamina papyracea (b, black arrow) it was not possible to get access to the originating site of tumor, and therefore ESS‐combined external approach surgery was employed to remove the tumor. Suppl. Figure 6c shows the eyebrow incision of the patient and Suppl. Figure 6d shows the endoscopic image of the tumor (black arrow and * indicated tumor and the most distal part of frontal sinus) from the external incision; with no recurrence of the tumor to date.
