# Supplementary figures and images for: Origin site‐based staging system of sinonasal inverted papilloma for application to endoscopic sinus surgery
Source: Head Neck. 2018 Dec 15;41(2):440–7. doi: 10.1002/hed.25435 (PMC6590184; doi:10.1002/hed.25435)

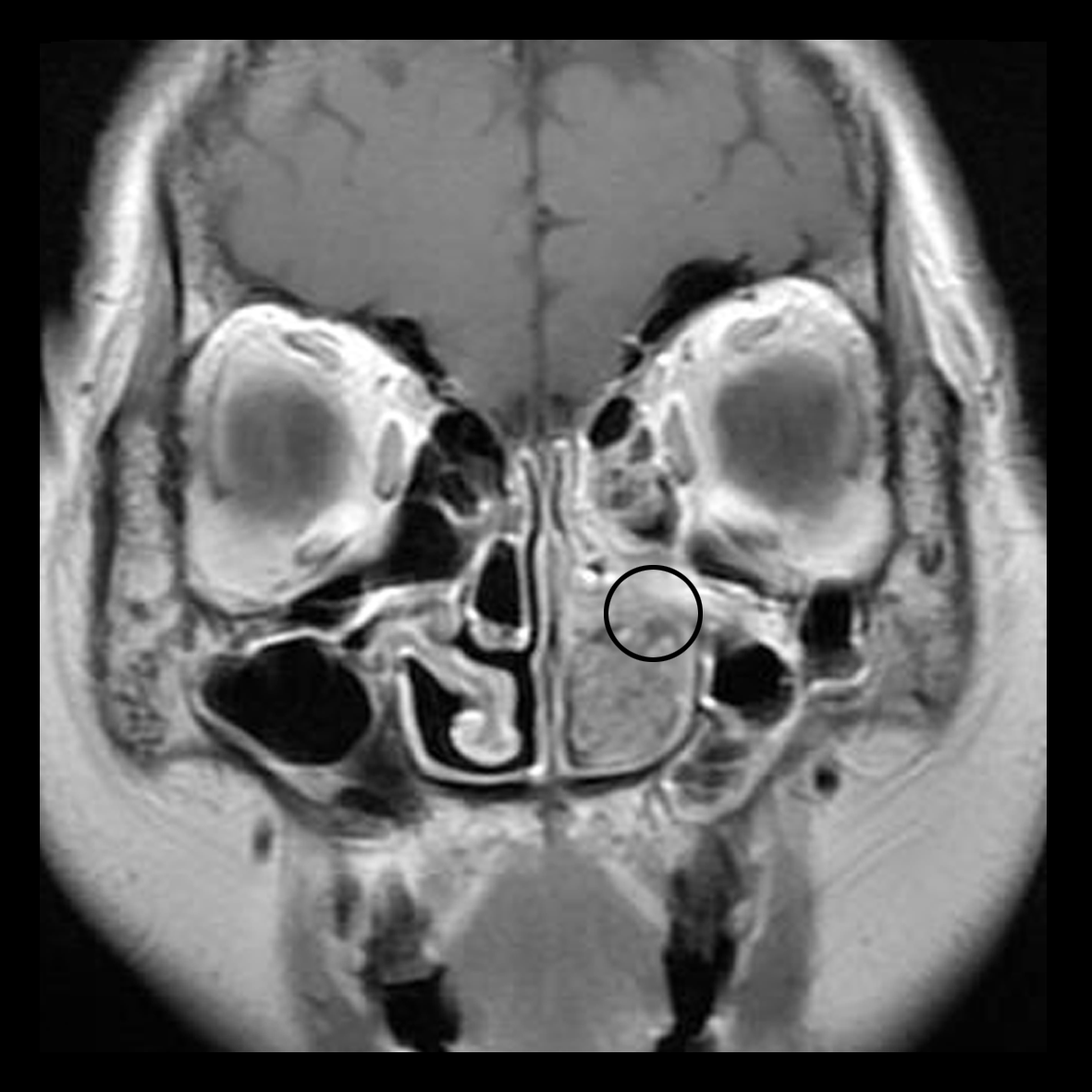

Supplement: Supplementary file 1 — Figure S1 Coronal contrast‐enhanced T1‐weighted MRI image (a) and nasal endoscopic examination (b) of a 45 years old woman, showing sinonasal inverted papilloma originating from left inferior turbinate (a, black circle). Endoscopic sinus surgery (ESS) was performed to remove the tumor, and 6 years after left inferior turbinate resection, there is no recurrence to date, as indicated by absence of tumor by CT scan (c) and endoscopy (d). Figure S2 Axial CT scan (a) and contrast‐enhanced T1‐weighted MRI image (b) of a 55 years old male, showing sinonasal inverted papilloma originating from posterior wall of left maxillary sinus (a, black circle; b, white circle). Endoscopic sinus surgery (ESS) was performed to remove the originating site of the tumor under 70° endoscopy (c ‐ tumor indicated by black arrow; d ‐ the tumor origin site after burning); with no recurrence to date 3 years after endoscopic sinus surgery. Figure S3 Axial (a) and coronal (b) contrast‐enhanced T1‐weighted MRI image of a 49 years old male, showing sinonasal inverted papilloma originating from anterior wall of left maxillary sinus (white circles). Endoscopic sinus surgery (ESS)‐assisted prelacrimal duct approach surgery was performed to remove the tumor (c ‐ black arrow indicates the originating site of the tumor, the white dotted lines indicated the lacrimal duct). Suppl. Figure 3d shows the origin site under 70° endoscopy after tumor resection (white dotted circle), A, I, M represented anterior, inferior, and medial wall of the maxillary sinus respectively; with no tumor recurrence after 5 years' follow‐up. Figure S4 Coronal CT scan (a) and contrast‐enhanced T1‐weighted MRI image (b) of a 59 years old female, showing sinonasal inverted papilloma originating from sphenoid sinus septum and affecting bilateral sinus. Endoscopic sinus surgery (ESS)‐assisted sphenoidal rostrum process approach surgery was performed to remove the tumor (c shows the endoscopic image after sphenoid sinus septum resection [file HED-41-440-s001.zip › hed25435-sup-0001-FigureS1.tif]

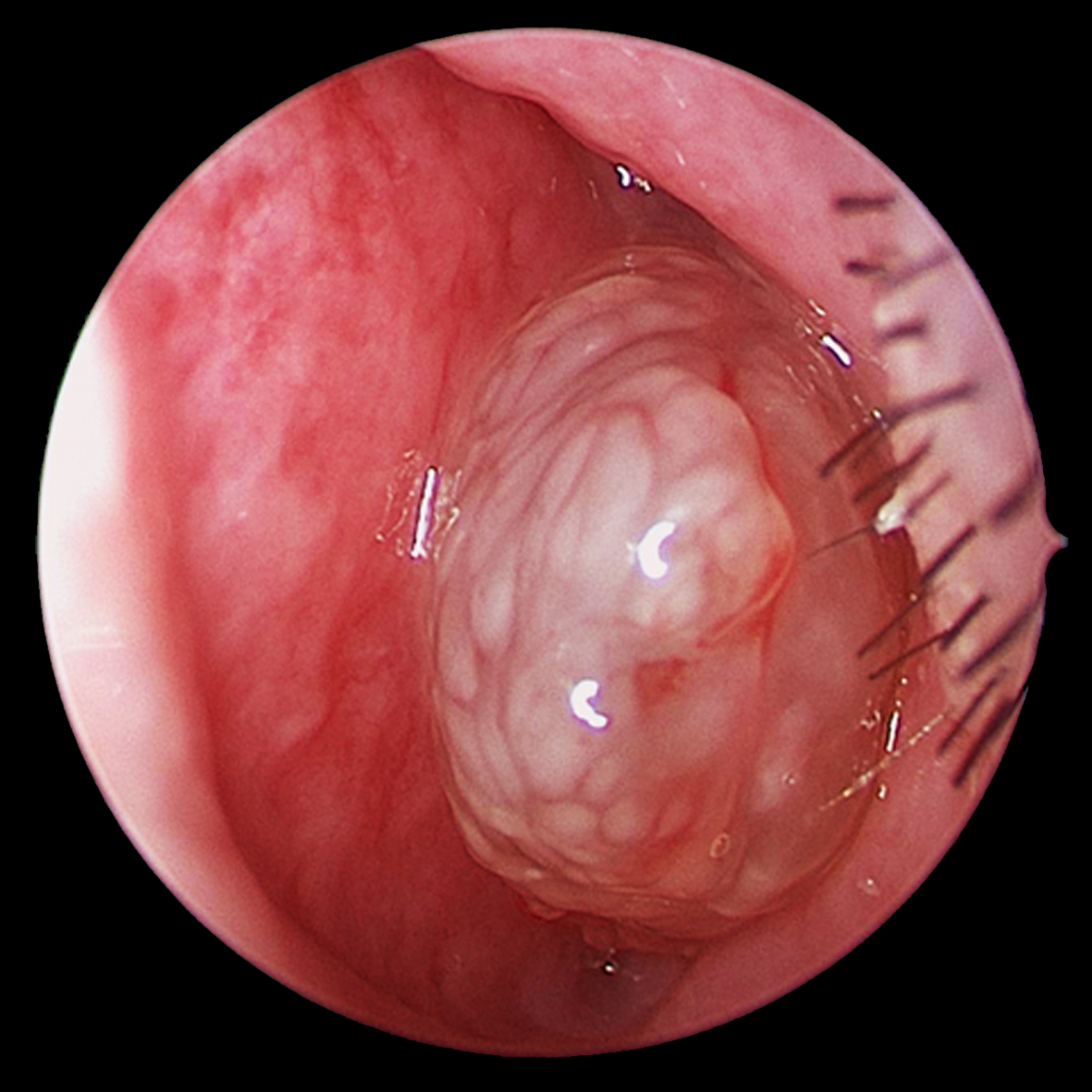

Supplement: Supplementary file 1 — Figure S1 Coronal contrast‐enhanced T1‐weighted MRI image (a) and nasal endoscopic examination (b) of a 45 years old woman, showing sinonasal inverted papilloma originating from left inferior turbinate (a, black circle). Endoscopic sinus surgery (ESS) was performed to remove the tumor, and 6 years after left inferior turbinate resection, there is no recurrence to date, as indicated by absence of tumor by CT scan (c) and endoscopy (d). Figure S2 Axial CT scan (a) and contrast‐enhanced T1‐weighted MRI image (b) of a 55 years old male, showing sinonasal inverted papilloma originating from posterior wall of left maxillary sinus (a, black circle; b, white circle). Endoscopic sinus surgery (ESS) was performed to remove the originating site of the tumor under 70° endoscopy (c ‐ tumor indicated by black arrow; d ‐ the tumor origin site after burning); with no recurrence to date 3 years after endoscopic sinus surgery. Figure S3 Axial (a) and coronal (b) contrast‐enhanced T1‐weighted MRI image of a 49 years old male, showing sinonasal inverted papilloma originating from anterior wall of left maxillary sinus (white circles). Endoscopic sinus surgery (ESS)‐assisted prelacrimal duct approach surgery was performed to remove the tumor (c ‐ black arrow indicates the originating site of the tumor, the white dotted lines indicated the lacrimal duct). Suppl. Figure 3d shows the origin site under 70° endoscopy after tumor resection (white dotted circle), A, I, M represented anterior, inferior, and medial wall of the maxillary sinus respectively; with no tumor recurrence after 5 years' follow‐up. Figure S4 Coronal CT scan (a) and contrast‐enhanced T1‐weighted MRI image (b) of a 59 years old female, showing sinonasal inverted papilloma originating from sphenoid sinus septum and affecting bilateral sinus. Endoscopic sinus surgery (ESS)‐assisted sphenoidal rostrum process approach surgery was performed to remove the tumor (c shows the endoscopic image after sphenoid sinus septum resection [file HED-41-440-s001.zip › hed25435-sup-0001-FigureS1a.tif]

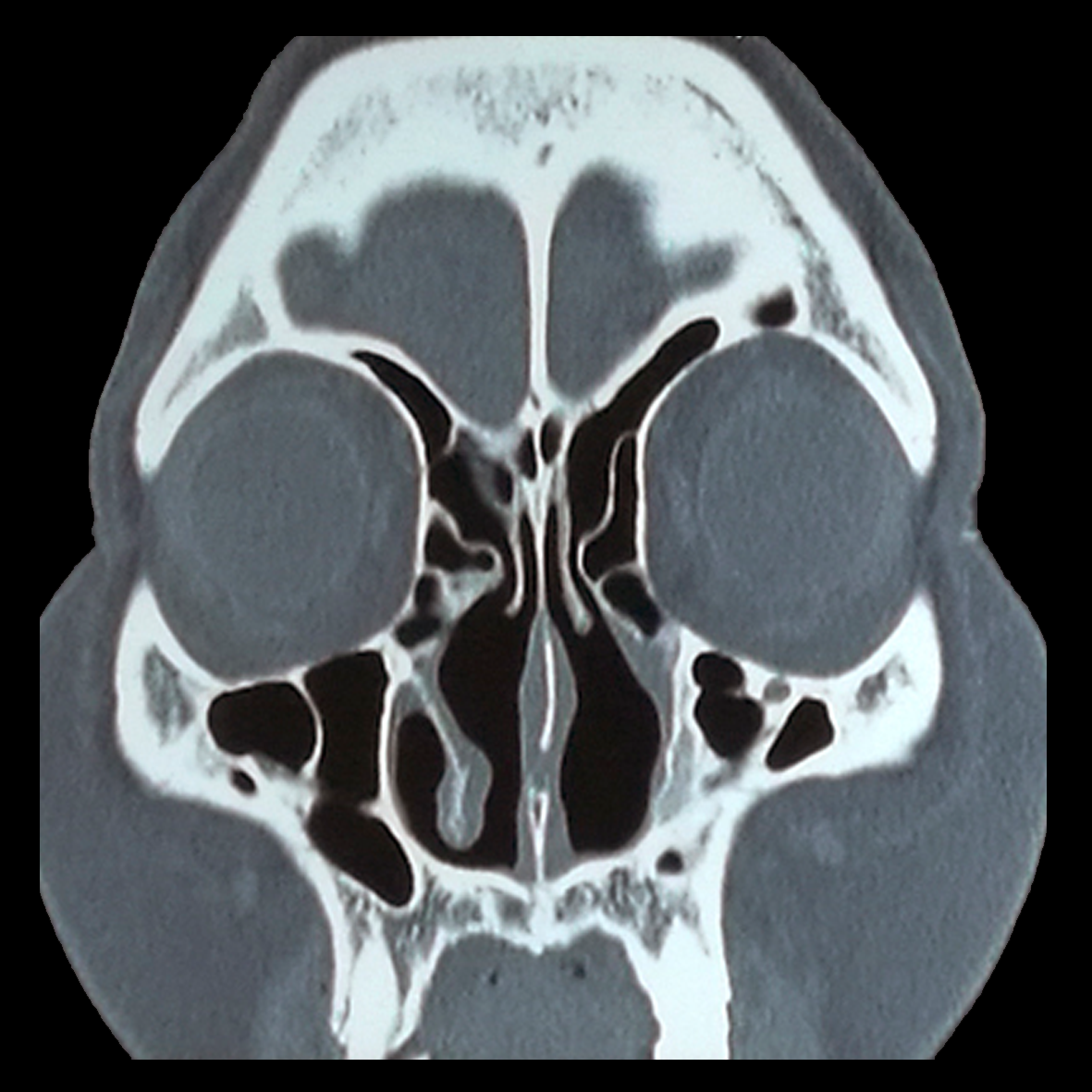

Supplement: Supplementary file 1 — Figure S1 Coronal contrast‐enhanced T1‐weighted MRI image (a) and nasal endoscopic examination (b) of a 45 years old woman, showing sinonasal inverted papilloma originating from left inferior turbinate (a, black circle). Endoscopic sinus surgery (ESS) was performed to remove the tumor, and 6 years after left inferior turbinate resection, there is no recurrence to date, as indicated by absence of tumor by CT scan (c) and endoscopy (d). Figure S2 Axial CT scan (a) and contrast‐enhanced T1‐weighted MRI image (b) of a 55 years old male, showing sinonasal inverted papilloma originating from posterior wall of left maxillary sinus (a, black circle; b, white circle). Endoscopic sinus surgery (ESS) was performed to remove the originating site of the tumor under 70° endoscopy (c ‐ tumor indicated by black arrow; d ‐ the tumor origin site after burning); with no recurrence to date 3 years after endoscopic sinus surgery. Figure S3 Axial (a) and coronal (b) contrast‐enhanced T1‐weighted MRI image of a 49 years old male, showing sinonasal inverted papilloma originating from anterior wall of left maxillary sinus (white circles). Endoscopic sinus surgery (ESS)‐assisted prelacrimal duct approach surgery was performed to remove the tumor (c ‐ black arrow indicates the originating site of the tumor, the white dotted lines indicated the lacrimal duct). Suppl. Figure 3d shows the origin site under 70° endoscopy after tumor resection (white dotted circle), A, I, M represented anterior, inferior, and medial wall of the maxillary sinus respectively; with no tumor recurrence after 5 years' follow‐up. Figure S4 Coronal CT scan (a) and contrast‐enhanced T1‐weighted MRI image (b) of a 59 years old female, showing sinonasal inverted papilloma originating from sphenoid sinus septum and affecting bilateral sinus. Endoscopic sinus surgery (ESS)‐assisted sphenoidal rostrum process approach surgery was performed to remove the tumor (c shows the endoscopic image after sphenoid sinus septum resection [file HED-41-440-s001.zip › hed25435-sup-0001-FigureS1b.tif]

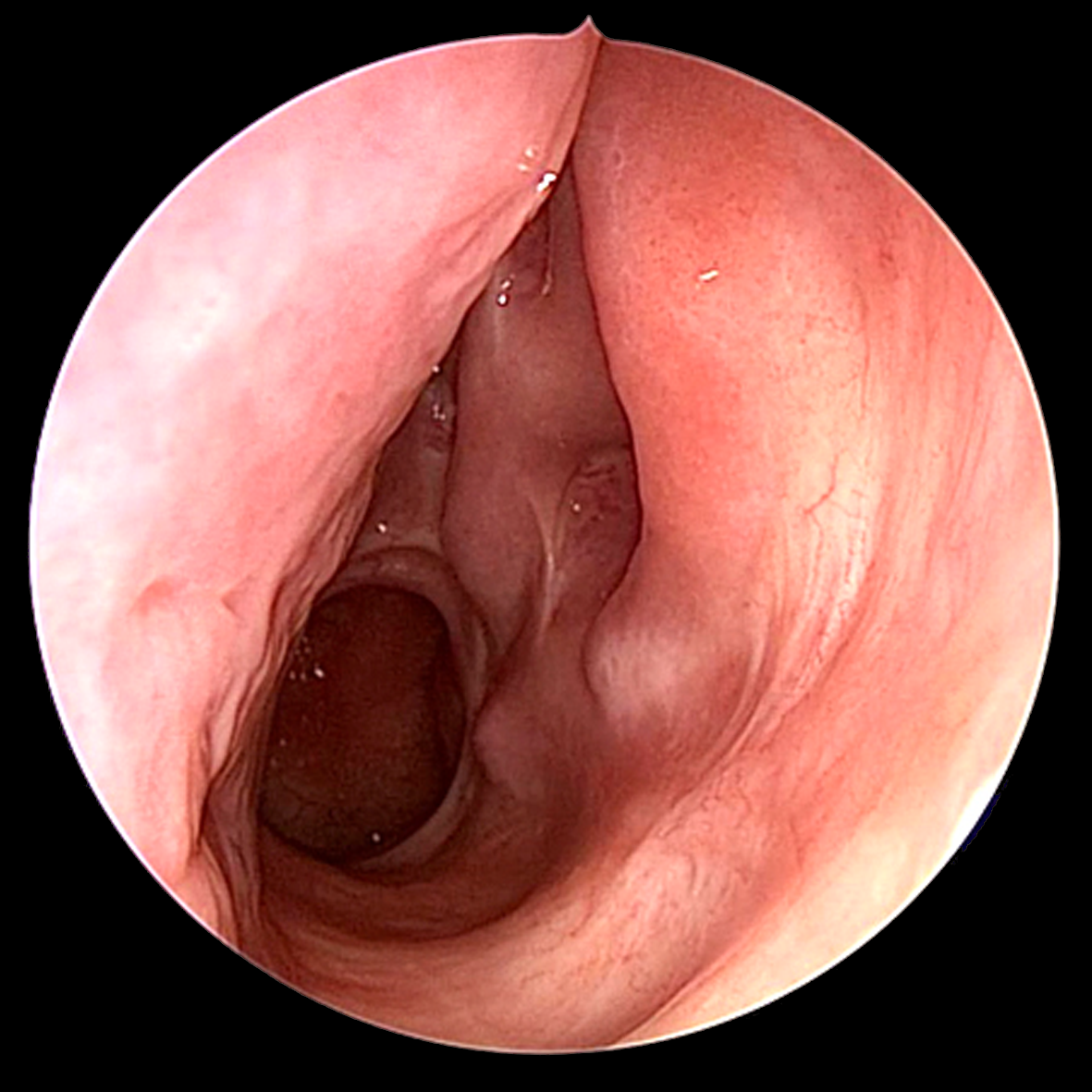

Supplement: Supplementary file 1 — Figure S1 Coronal contrast‐enhanced T1‐weighted MRI image (a) and nasal endoscopic examination (b) of a 45 years old woman, showing sinonasal inverted papilloma originating from left inferior turbinate (a, black circle). Endoscopic sinus surgery (ESS) was performed to remove the tumor, and 6 years after left inferior turbinate resection, there is no recurrence to date, as indicated by absence of tumor by CT scan (c) and endoscopy (d). Figure S2 Axial CT scan (a) and contrast‐enhanced T1‐weighted MRI image (b) of a 55 years old male, showing sinonasal inverted papilloma originating from posterior wall of left maxillary sinus (a, black circle; b, white circle). Endoscopic sinus surgery (ESS) was performed to remove the originating site of the tumor under 70° endoscopy (c ‐ tumor indicated by black arrow; d ‐ the tumor origin site after burning); with no recurrence to date 3 years after endoscopic sinus surgery. Figure S3 Axial (a) and coronal (b) contrast‐enhanced T1‐weighted MRI image of a 49 years old male, showing sinonasal inverted papilloma originating from anterior wall of left maxillary sinus (white circles). Endoscopic sinus surgery (ESS)‐assisted prelacrimal duct approach surgery was performed to remove the tumor (c ‐ black arrow indicates the originating site of the tumor, the white dotted lines indicated the lacrimal duct). Suppl. Figure 3d shows the origin site under 70° endoscopy after tumor resection (white dotted circle), A, I, M represented anterior, inferior, and medial wall of the maxillary sinus respectively; with no tumor recurrence after 5 years' follow‐up. Figure S4 Coronal CT scan (a) and contrast‐enhanced T1‐weighted MRI image (b) of a 59 years old female, showing sinonasal inverted papilloma originating from sphenoid sinus septum and affecting bilateral sinus. Endoscopic sinus surgery (ESS)‐assisted sphenoidal rostrum process approach surgery was performed to remove the tumor (c shows the endoscopic image after sphenoid sinus septum resection [file HED-41-440-s001.zip › hed25435-sup-0001-FigureS1c.tif]

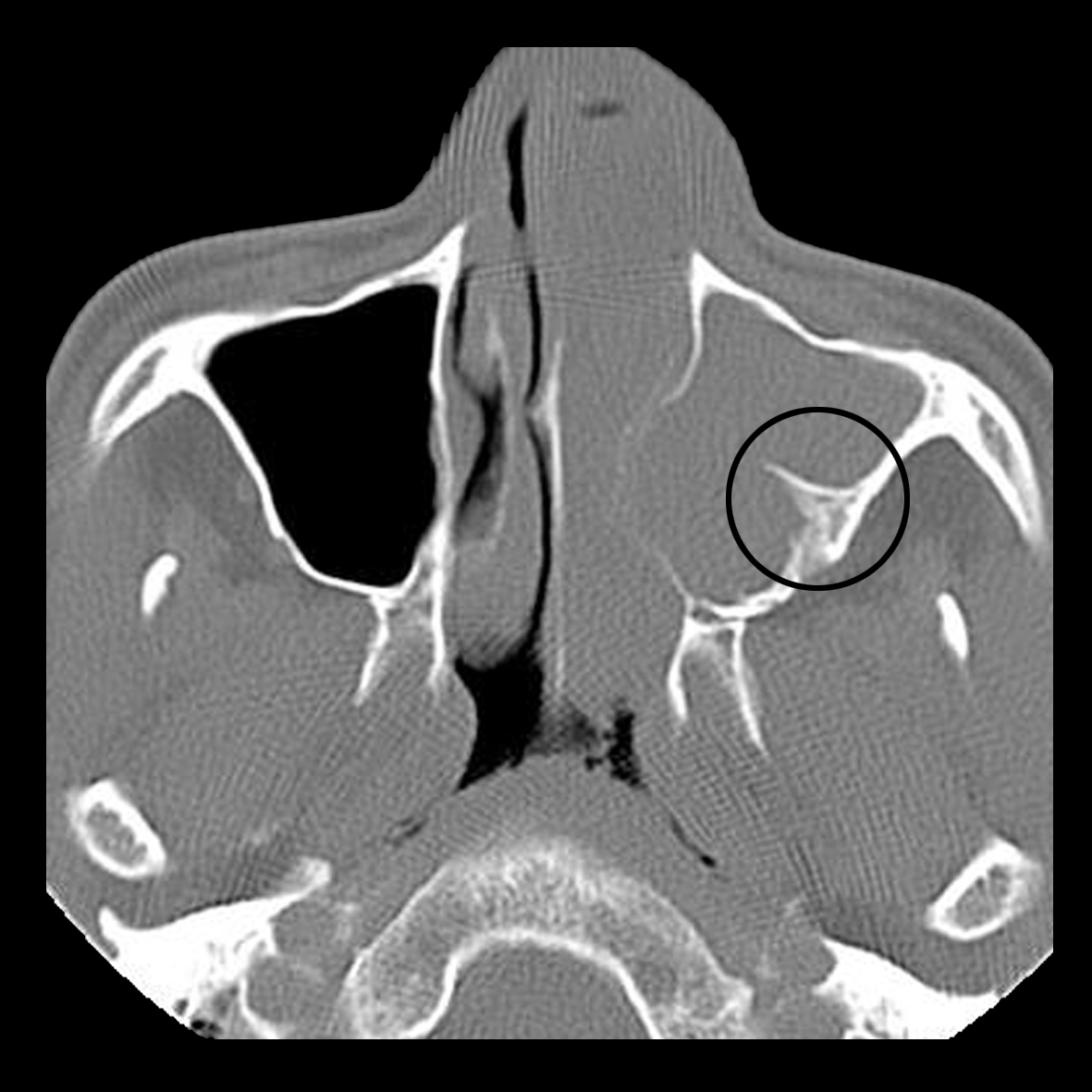

Supplement: Supplementary file 1 — Figure S1 Coronal contrast‐enhanced T1‐weighted MRI image (a) and nasal endoscopic examination (b) of a 45 years old woman, showing sinonasal inverted papilloma originating from left inferior turbinate (a, black circle). Endoscopic sinus surgery (ESS) was performed to remove the tumor, and 6 years after left inferior turbinate resection, there is no recurrence to date, as indicated by absence of tumor by CT scan (c) and endoscopy (d). Figure S2 Axial CT scan (a) and contrast‐enhanced T1‐weighted MRI image (b) of a 55 years old male, showing sinonasal inverted papilloma originating from posterior wall of left maxillary sinus (a, black circle; b, white circle). Endoscopic sinus surgery (ESS) was performed to remove the originating site of the tumor under 70° endoscopy (c ‐ tumor indicated by black arrow; d ‐ the tumor origin site after burning); with no recurrence to date 3 years after endoscopic sinus surgery. Figure S3 Axial (a) and coronal (b) contrast‐enhanced T1‐weighted MRI image of a 49 years old male, showing sinonasal inverted papilloma originating from anterior wall of left maxillary sinus (white circles). Endoscopic sinus surgery (ESS)‐assisted prelacrimal duct approach surgery was performed to remove the tumor (c ‐ black arrow indicates the originating site of the tumor, the white dotted lines indicated the lacrimal duct). Suppl. Figure 3d shows the origin site under 70° endoscopy after tumor resection (white dotted circle), A, I, M represented anterior, inferior, and medial wall of the maxillary sinus respectively; with no tumor recurrence after 5 years' follow‐up. Figure S4 Coronal CT scan (a) and contrast‐enhanced T1‐weighted MRI image (b) of a 59 years old female, showing sinonasal inverted papilloma originating from sphenoid sinus septum and affecting bilateral sinus. Endoscopic sinus surgery (ESS)‐assisted sphenoidal rostrum process approach surgery was performed to remove the tumor (c shows the endoscopic image after sphenoid sinus septum resection [file HED-41-440-s001.zip › hed25435-sup-0002-FigureS2.tif]

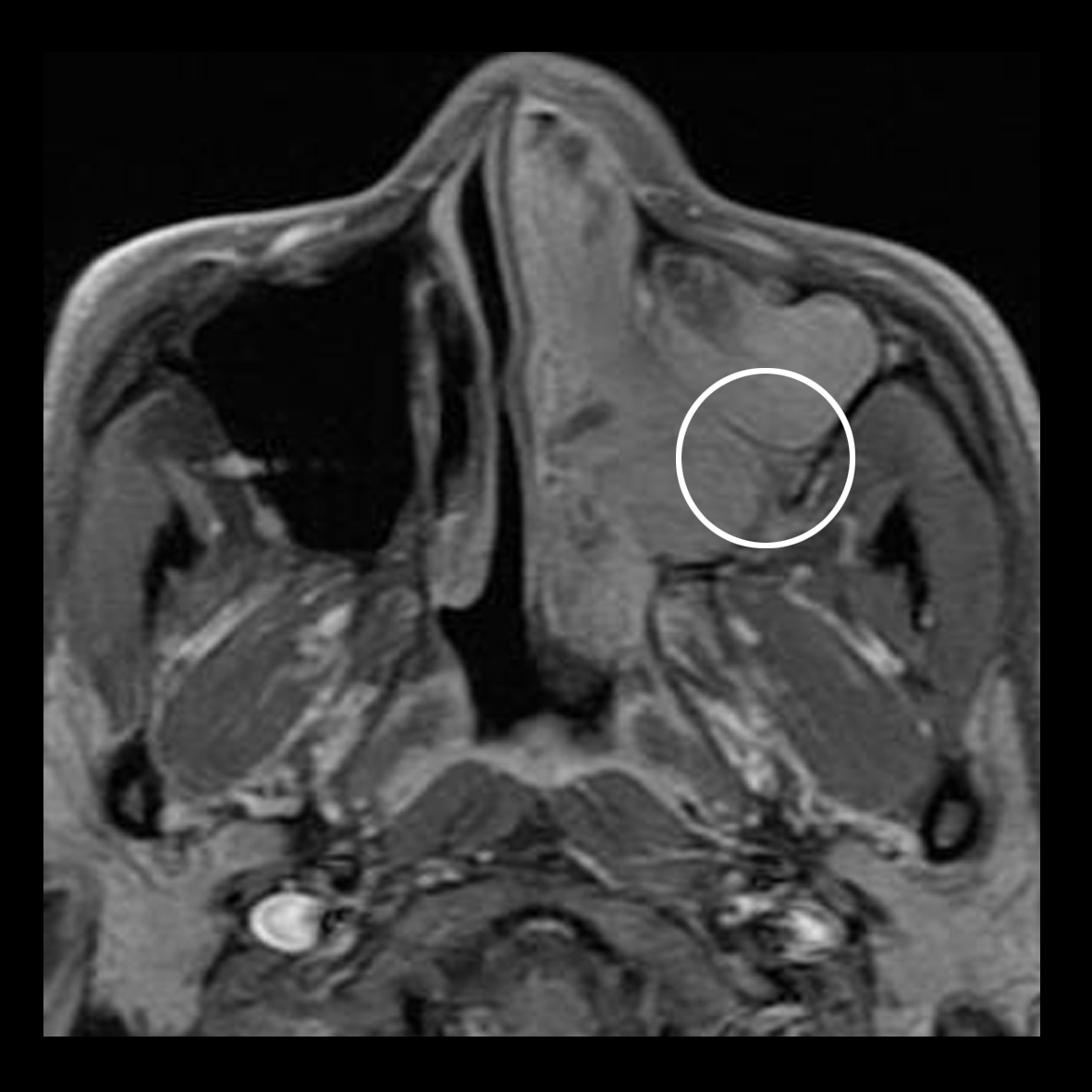

Supplement: Supplementary file 1 — Figure S1 Coronal contrast‐enhanced T1‐weighted MRI image (a) and nasal endoscopic examination (b) of a 45 years old woman, showing sinonasal inverted papilloma originating from left inferior turbinate (a, black circle). Endoscopic sinus surgery (ESS) was performed to remove the tumor, and 6 years after left inferior turbinate resection, there is no recurrence to date, as indicated by absence of tumor by CT scan (c) and endoscopy (d). Figure S2 Axial CT scan (a) and contrast‐enhanced T1‐weighted MRI image (b) of a 55 years old male, showing sinonasal inverted papilloma originating from posterior wall of left maxillary sinus (a, black circle; b, white circle). Endoscopic sinus surgery (ESS) was performed to remove the originating site of the tumor under 70° endoscopy (c ‐ tumor indicated by black arrow; d ‐ the tumor origin site after burning); with no recurrence to date 3 years after endoscopic sinus surgery. Figure S3 Axial (a) and coronal (b) contrast‐enhanced T1‐weighted MRI image of a 49 years old male, showing sinonasal inverted papilloma originating from anterior wall of left maxillary sinus (white circles). Endoscopic sinus surgery (ESS)‐assisted prelacrimal duct approach surgery was performed to remove the tumor (c ‐ black arrow indicates the originating site of the tumor, the white dotted lines indicated the lacrimal duct). Suppl. Figure 3d shows the origin site under 70° endoscopy after tumor resection (white dotted circle), A, I, M represented anterior, inferior, and medial wall of the maxillary sinus respectively; with no tumor recurrence after 5 years' follow‐up. Figure S4 Coronal CT scan (a) and contrast‐enhanced T1‐weighted MRI image (b) of a 59 years old female, showing sinonasal inverted papilloma originating from sphenoid sinus septum and affecting bilateral sinus. Endoscopic sinus surgery (ESS)‐assisted sphenoidal rostrum process approach surgery was performed to remove the tumor (c shows the endoscopic image after sphenoid sinus septum resection [file HED-41-440-s001.zip › hed25435-sup-0002-FigureS2a.tif]

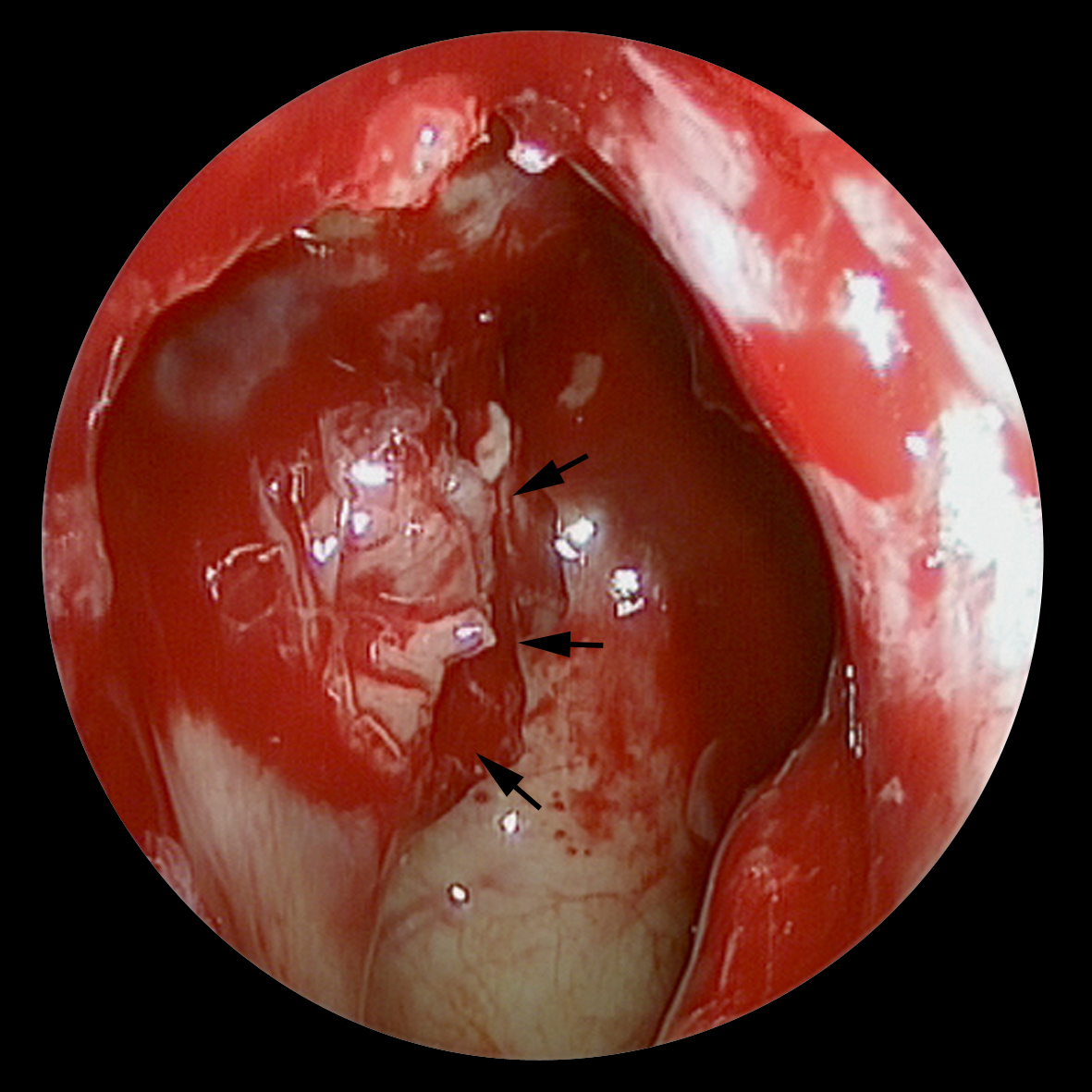

Supplement: Supplementary file 1 — Figure S1 Coronal contrast‐enhanced T1‐weighted MRI image (a) and nasal endoscopic examination (b) of a 45 years old woman, showing sinonasal inverted papilloma originating from left inferior turbinate (a, black circle). Endoscopic sinus surgery (ESS) was performed to remove the tumor, and 6 years after left inferior turbinate resection, there is no recurrence to date, as indicated by absence of tumor by CT scan (c) and endoscopy (d). Figure S2 Axial CT scan (a) and contrast‐enhanced T1‐weighted MRI image (b) of a 55 years old male, showing sinonasal inverted papilloma originating from posterior wall of left maxillary sinus (a, black circle; b, white circle). Endoscopic sinus surgery (ESS) was performed to remove the originating site of the tumor under 70° endoscopy (c ‐ tumor indicated by black arrow; d ‐ the tumor origin site after burning); with no recurrence to date 3 years after endoscopic sinus surgery. Figure S3 Axial (a) and coronal (b) contrast‐enhanced T1‐weighted MRI image of a 49 years old male, showing sinonasal inverted papilloma originating from anterior wall of left maxillary sinus (white circles). Endoscopic sinus surgery (ESS)‐assisted prelacrimal duct approach surgery was performed to remove the tumor (c ‐ black arrow indicates the originating site of the tumor, the white dotted lines indicated the lacrimal duct). Suppl. Figure 3d shows the origin site under 70° endoscopy after tumor resection (white dotted circle), A, I, M represented anterior, inferior, and medial wall of the maxillary sinus respectively; with no tumor recurrence after 5 years' follow‐up. Figure S4 Coronal CT scan (a) and contrast‐enhanced T1‐weighted MRI image (b) of a 59 years old female, showing sinonasal inverted papilloma originating from sphenoid sinus septum and affecting bilateral sinus. Endoscopic sinus surgery (ESS)‐assisted sphenoidal rostrum process approach surgery was performed to remove the tumor (c shows the endoscopic image after sphenoid sinus septum resection [file HED-41-440-s001.zip › hed25435-sup-0002-FigureS2b.tif]

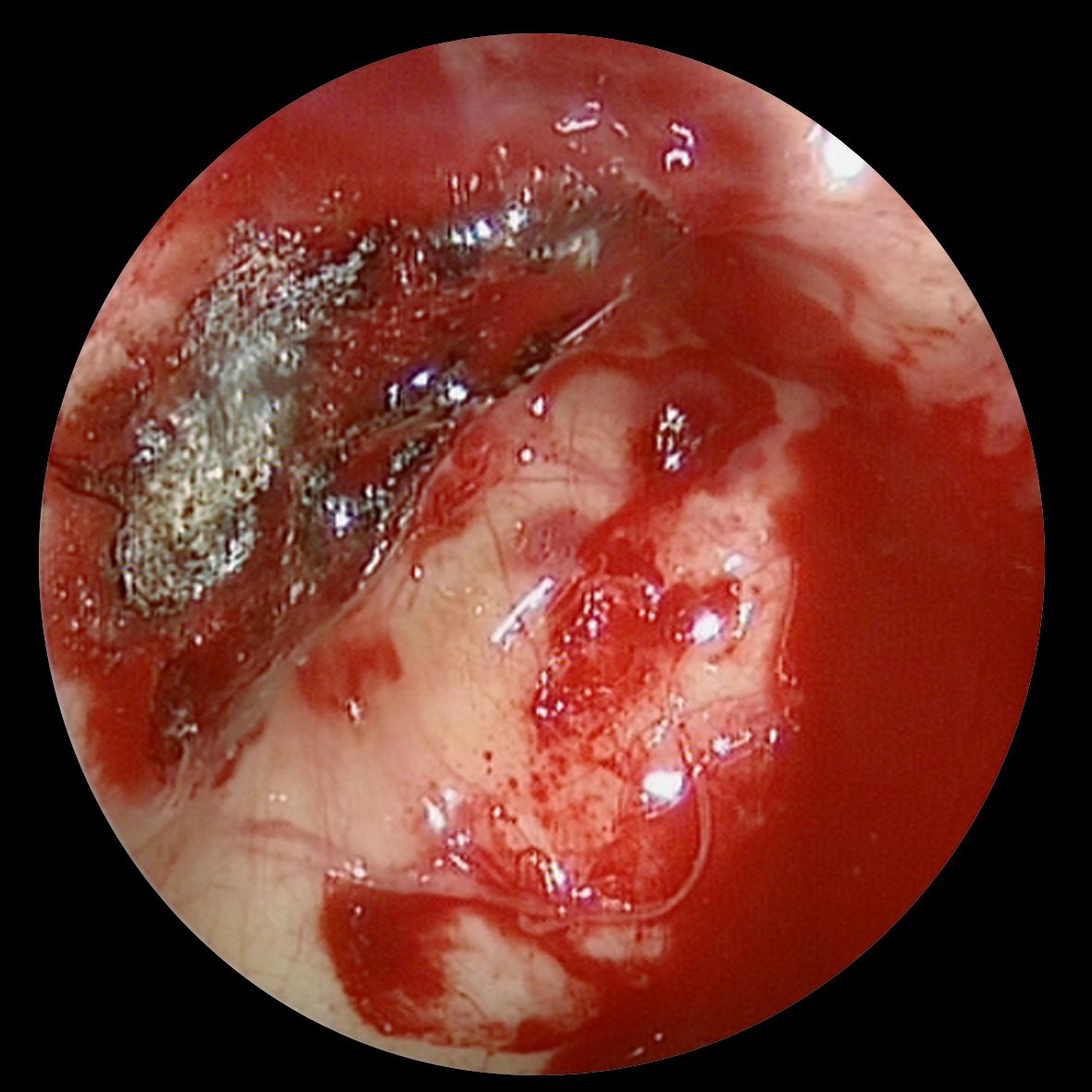

Supplement: Supplementary file 1 — Figure S1 Coronal contrast‐enhanced T1‐weighted MRI image (a) and nasal endoscopic examination (b) of a 45 years old woman, showing sinonasal inverted papilloma originating from left inferior turbinate (a, black circle). Endoscopic sinus surgery (ESS) was performed to remove the tumor, and 6 years after left inferior turbinate resection, there is no recurrence to date, as indicated by absence of tumor by CT scan (c) and endoscopy (d). Figure S2 Axial CT scan (a) and contrast‐enhanced T1‐weighted MRI image (b) of a 55 years old male, showing sinonasal inverted papilloma originating from posterior wall of left maxillary sinus (a, black circle; b, white circle). Endoscopic sinus surgery (ESS) was performed to remove the originating site of the tumor under 70° endoscopy (c ‐ tumor indicated by black arrow; d ‐ the tumor origin site after burning); with no recurrence to date 3 years after endoscopic sinus surgery. Figure S3 Axial (a) and coronal (b) contrast‐enhanced T1‐weighted MRI image of a 49 years old male, showing sinonasal inverted papilloma originating from anterior wall of left maxillary sinus (white circles). Endoscopic sinus surgery (ESS)‐assisted prelacrimal duct approach surgery was performed to remove the tumor (c ‐ black arrow indicates the originating site of the tumor, the white dotted lines indicated the lacrimal duct). Suppl. Figure 3d shows the origin site under 70° endoscopy after tumor resection (white dotted circle), A, I, M represented anterior, inferior, and medial wall of the maxillary sinus respectively; with no tumor recurrence after 5 years' follow‐up. Figure S4 Coronal CT scan (a) and contrast‐enhanced T1‐weighted MRI image (b) of a 59 years old female, showing sinonasal inverted papilloma originating from sphenoid sinus septum and affecting bilateral sinus. Endoscopic sinus surgery (ESS)‐assisted sphenoidal rostrum process approach surgery was performed to remove the tumor (c shows the endoscopic image after sphenoid sinus septum resection [file HED-41-440-s001.zip › hed25435-sup-0002-FigureS2c.tif]

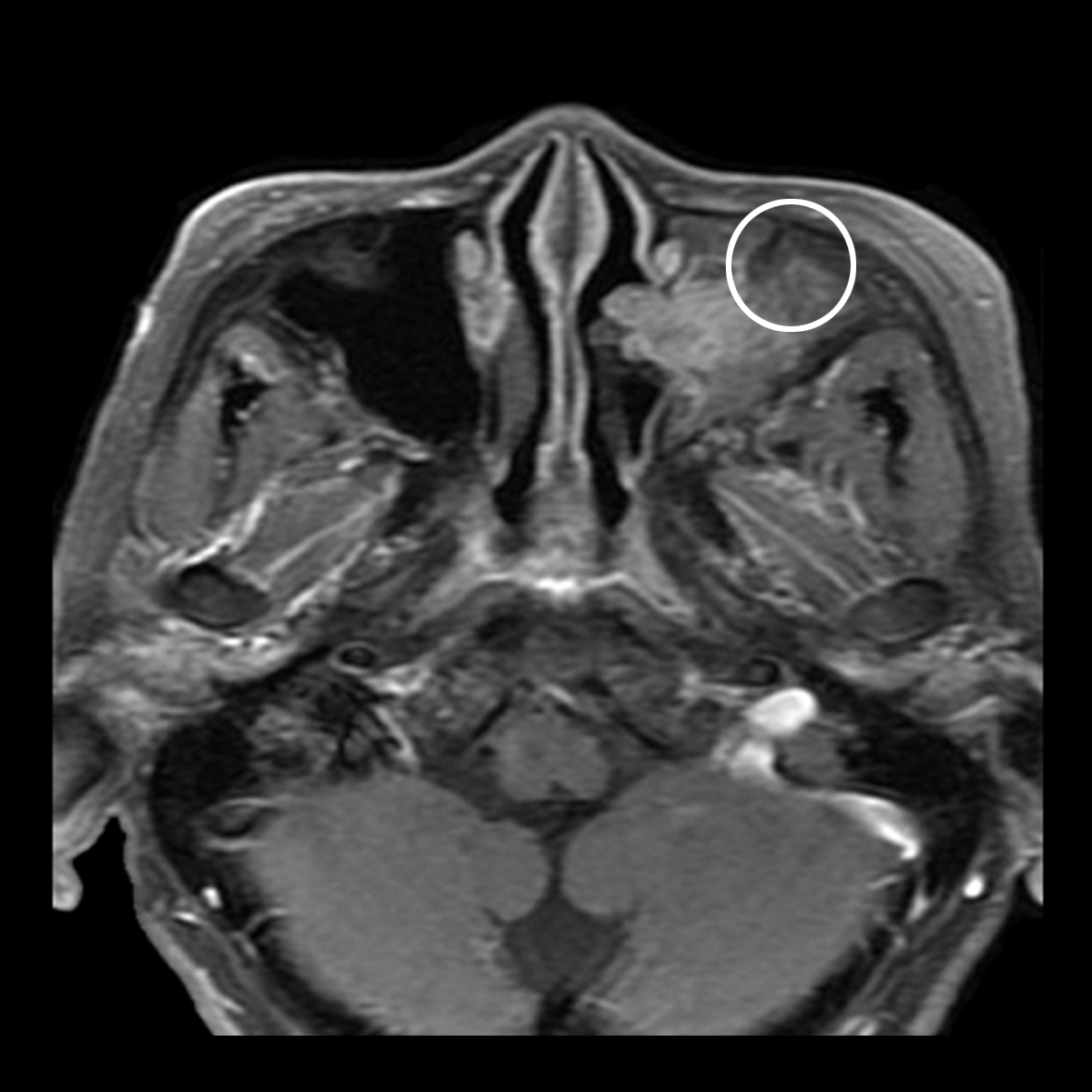

Supplement: Supplementary file 1 — Figure S1 Coronal contrast‐enhanced T1‐weighted MRI image (a) and nasal endoscopic examination (b) of a 45 years old woman, showing sinonasal inverted papilloma originating from left inferior turbinate (a, black circle). Endoscopic sinus surgery (ESS) was performed to remove the tumor, and 6 years after left inferior turbinate resection, there is no recurrence to date, as indicated by absence of tumor by CT scan (c) and endoscopy (d). Figure S2 Axial CT scan (a) and contrast‐enhanced T1‐weighted MRI image (b) of a 55 years old male, showing sinonasal inverted papilloma originating from posterior wall of left maxillary sinus (a, black circle; b, white circle). Endoscopic sinus surgery (ESS) was performed to remove the originating site of the tumor under 70° endoscopy (c ‐ tumor indicated by black arrow; d ‐ the tumor origin site after burning); with no recurrence to date 3 years after endoscopic sinus surgery. Figure S3 Axial (a) and coronal (b) contrast‐enhanced T1‐weighted MRI image of a 49 years old male, showing sinonasal inverted papilloma originating from anterior wall of left maxillary sinus (white circles). Endoscopic sinus surgery (ESS)‐assisted prelacrimal duct approach surgery was performed to remove the tumor (c ‐ black arrow indicates the originating site of the tumor, the white dotted lines indicated the lacrimal duct). Suppl. Figure 3d shows the origin site under 70° endoscopy after tumor resection (white dotted circle), A, I, M represented anterior, inferior, and medial wall of the maxillary sinus respectively; with no tumor recurrence after 5 years' follow‐up. Figure S4 Coronal CT scan (a) and contrast‐enhanced T1‐weighted MRI image (b) of a 59 years old female, showing sinonasal inverted papilloma originating from sphenoid sinus septum and affecting bilateral sinus. Endoscopic sinus surgery (ESS)‐assisted sphenoidal rostrum process approach surgery was performed to remove the tumor (c shows the endoscopic image after sphenoid sinus septum resection [file HED-41-440-s001.zip › hed25435-sup-0003-FigureS3.tif]

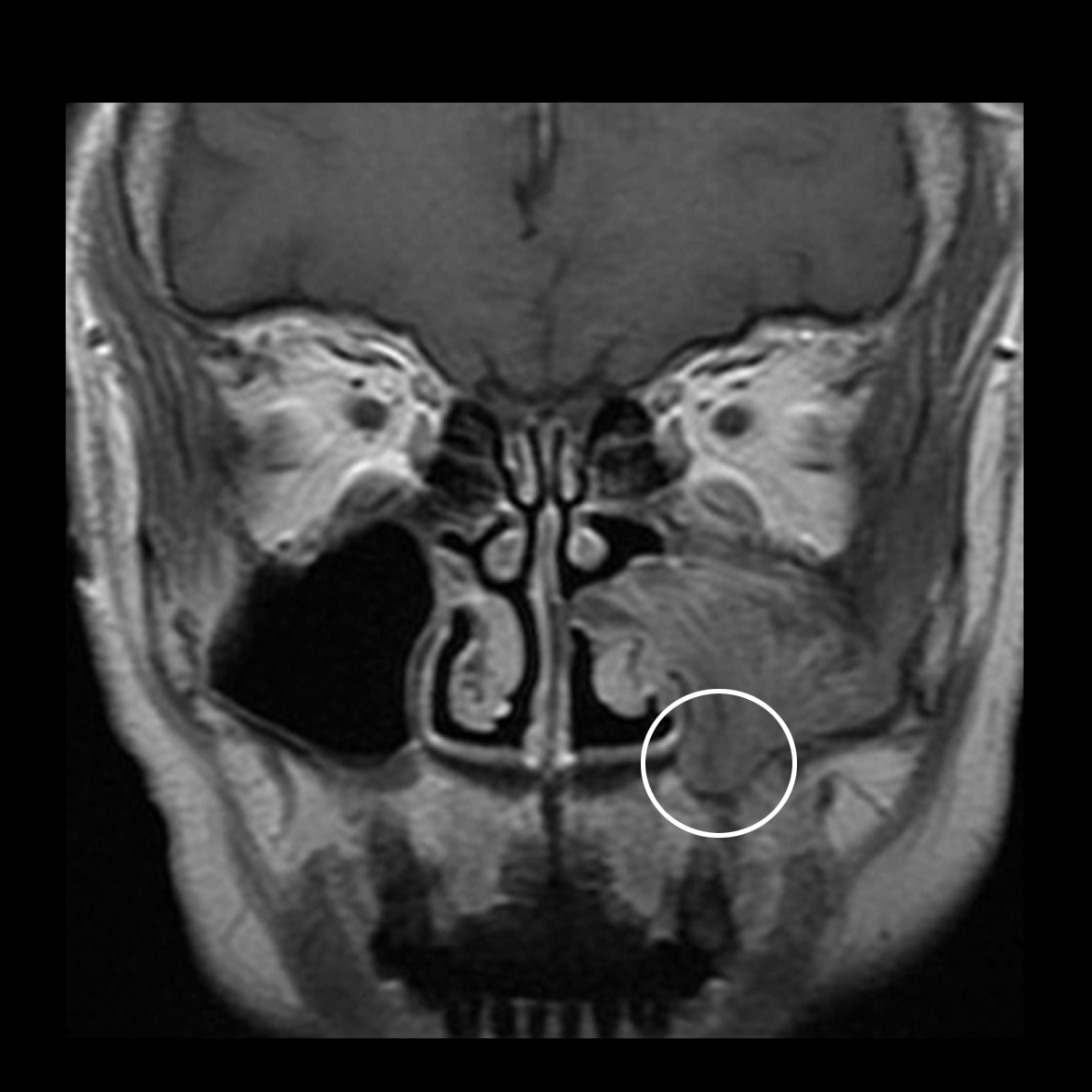

Supplement: Supplementary file 1 — Figure S1 Coronal contrast‐enhanced T1‐weighted MRI image (a) and nasal endoscopic examination (b) of a 45 years old woman, showing sinonasal inverted papilloma originating from left inferior turbinate (a, black circle). Endoscopic sinus surgery (ESS) was performed to remove the tumor, and 6 years after left inferior turbinate resection, there is no recurrence to date, as indicated by absence of tumor by CT scan (c) and endoscopy (d). Figure S2 Axial CT scan (a) and contrast‐enhanced T1‐weighted MRI image (b) of a 55 years old male, showing sinonasal inverted papilloma originating from posterior wall of left maxillary sinus (a, black circle; b, white circle). Endoscopic sinus surgery (ESS) was performed to remove the originating site of the tumor under 70° endoscopy (c ‐ tumor indicated by black arrow; d ‐ the tumor origin site after burning); with no recurrence to date 3 years after endoscopic sinus surgery. Figure S3 Axial (a) and coronal (b) contrast‐enhanced T1‐weighted MRI image of a 49 years old male, showing sinonasal inverted papilloma originating from anterior wall of left maxillary sinus (white circles). Endoscopic sinus surgery (ESS)‐assisted prelacrimal duct approach surgery was performed to remove the tumor (c ‐ black arrow indicates the originating site of the tumor, the white dotted lines indicated the lacrimal duct). Suppl. Figure 3d shows the origin site under 70° endoscopy after tumor resection (white dotted circle), A, I, M represented anterior, inferior, and medial wall of the maxillary sinus respectively; with no tumor recurrence after 5 years' follow‐up. Figure S4 Coronal CT scan (a) and contrast‐enhanced T1‐weighted MRI image (b) of a 59 years old female, showing sinonasal inverted papilloma originating from sphenoid sinus septum and affecting bilateral sinus. Endoscopic sinus surgery (ESS)‐assisted sphenoidal rostrum process approach surgery was performed to remove the tumor (c shows the endoscopic image after sphenoid sinus septum resection [file HED-41-440-s001.zip › hed25435-sup-0003-FigureS3a.tif]

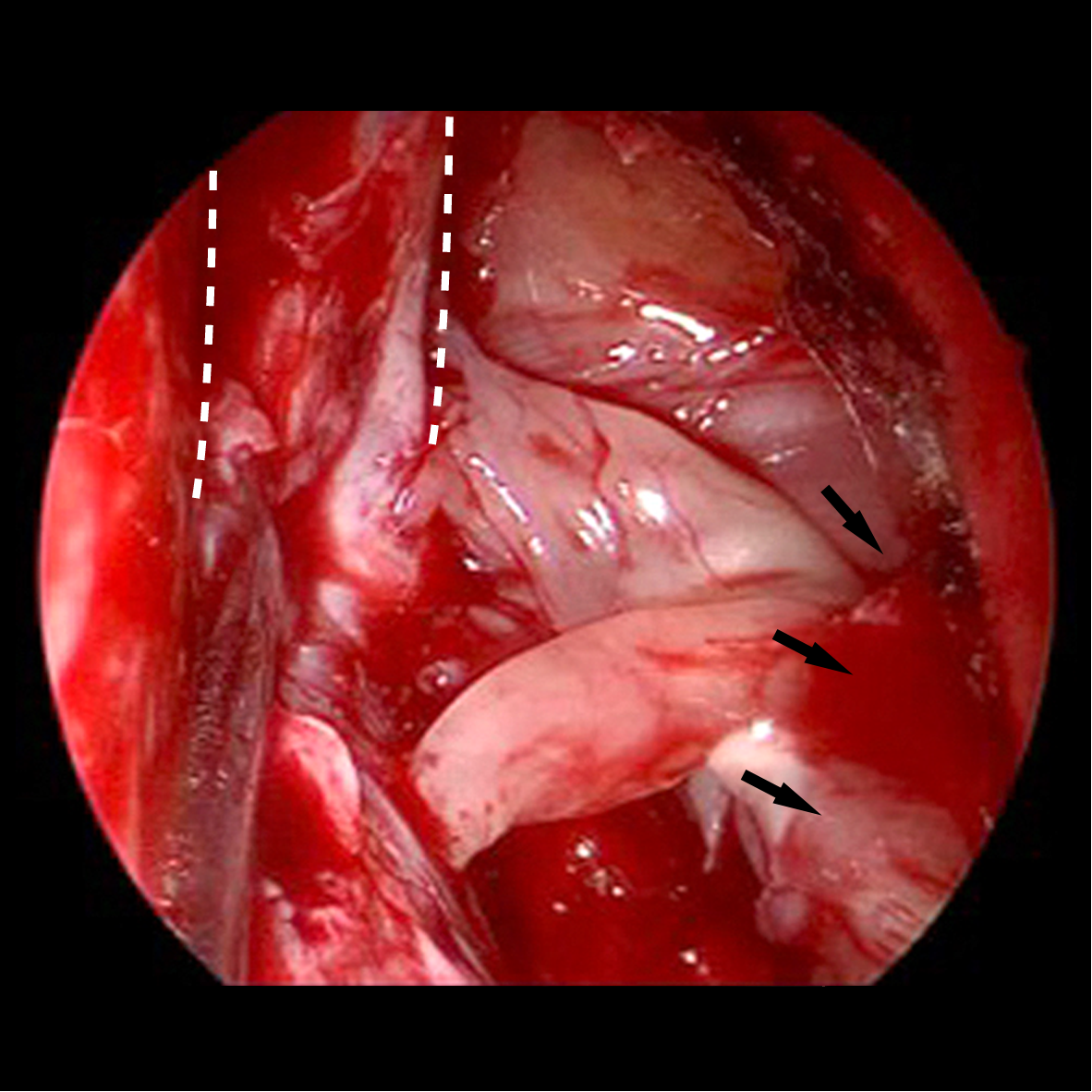

Supplement: Supplementary file 1 — Figure S1 Coronal contrast‐enhanced T1‐weighted MRI image (a) and nasal endoscopic examination (b) of a 45 years old woman, showing sinonasal inverted papilloma originating from left inferior turbinate (a, black circle). Endoscopic sinus surgery (ESS) was performed to remove the tumor, and 6 years after left inferior turbinate resection, there is no recurrence to date, as indicated by absence of tumor by CT scan (c) and endoscopy (d). Figure S2 Axial CT scan (a) and contrast‐enhanced T1‐weighted MRI image (b) of a 55 years old male, showing sinonasal inverted papilloma originating from posterior wall of left maxillary sinus (a, black circle; b, white circle). Endoscopic sinus surgery (ESS) was performed to remove the originating site of the tumor under 70° endoscopy (c ‐ tumor indicated by black arrow; d ‐ the tumor origin site after burning); with no recurrence to date 3 years after endoscopic sinus surgery. Figure S3 Axial (a) and coronal (b) contrast‐enhanced T1‐weighted MRI image of a 49 years old male, showing sinonasal inverted papilloma originating from anterior wall of left maxillary sinus (white circles). Endoscopic sinus surgery (ESS)‐assisted prelacrimal duct approach surgery was performed to remove the tumor (c ‐ black arrow indicates the originating site of the tumor, the white dotted lines indicated the lacrimal duct). Suppl. Figure 3d shows the origin site under 70° endoscopy after tumor resection (white dotted circle), A, I, M represented anterior, inferior, and medial wall of the maxillary sinus respectively; with no tumor recurrence after 5 years' follow‐up. Figure S4 Coronal CT scan (a) and contrast‐enhanced T1‐weighted MRI image (b) of a 59 years old female, showing sinonasal inverted papilloma originating from sphenoid sinus septum and affecting bilateral sinus. Endoscopic sinus surgery (ESS)‐assisted sphenoidal rostrum process approach surgery was performed to remove the tumor (c shows the endoscopic image after sphenoid sinus septum resection [file HED-41-440-s001.zip › hed25435-sup-0003-FigureS3b.tif]

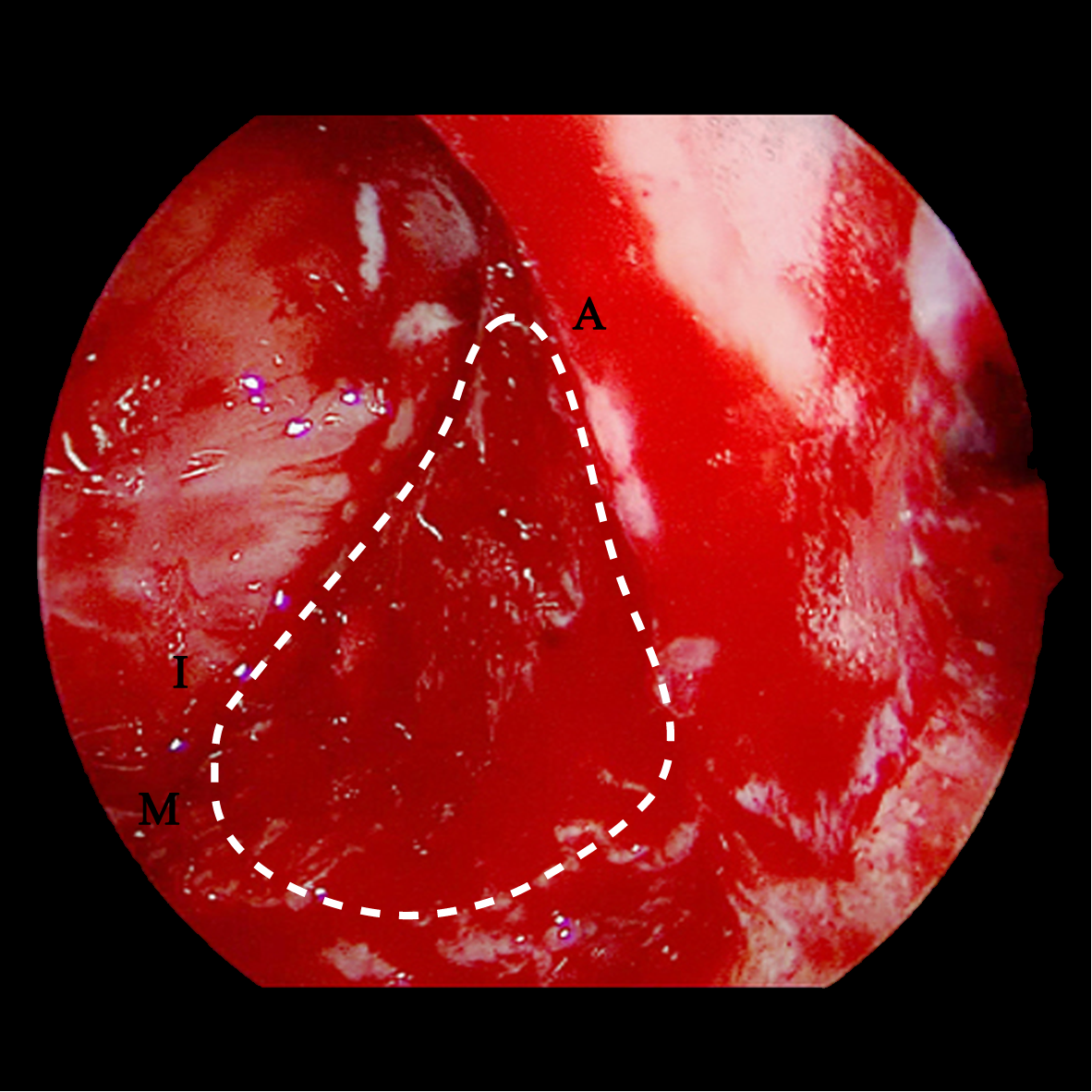

Supplement: Supplementary file 1 — Figure S1 Coronal contrast‐enhanced T1‐weighted MRI image (a) and nasal endoscopic examination (b) of a 45 years old woman, showing sinonasal inverted papilloma originating from left inferior turbinate (a, black circle). Endoscopic sinus surgery (ESS) was performed to remove the tumor, and 6 years after left inferior turbinate resection, there is no recurrence to date, as indicated by absence of tumor by CT scan (c) and endoscopy (d). Figure S2 Axial CT scan (a) and contrast‐enhanced T1‐weighted MRI image (b) of a 55 years old male, showing sinonasal inverted papilloma originating from posterior wall of left maxillary sinus (a, black circle; b, white circle). Endoscopic sinus surgery (ESS) was performed to remove the originating site of the tumor under 70° endoscopy (c ‐ tumor indicated by black arrow; d ‐ the tumor origin site after burning); with no recurrence to date 3 years after endoscopic sinus surgery. Figure S3 Axial (a) and coronal (b) contrast‐enhanced T1‐weighted MRI image of a 49 years old male, showing sinonasal inverted papilloma originating from anterior wall of left maxillary sinus (white circles). Endoscopic sinus surgery (ESS)‐assisted prelacrimal duct approach surgery was performed to remove the tumor (c ‐ black arrow indicates the originating site of the tumor, the white dotted lines indicated the lacrimal duct). Suppl. Figure 3d shows the origin site under 70° endoscopy after tumor resection (white dotted circle), A, I, M represented anterior, inferior, and medial wall of the maxillary sinus respectively; with no tumor recurrence after 5 years' follow‐up. Figure S4 Coronal CT scan (a) and contrast‐enhanced T1‐weighted MRI image (b) of a 59 years old female, showing sinonasal inverted papilloma originating from sphenoid sinus septum and affecting bilateral sinus. Endoscopic sinus surgery (ESS)‐assisted sphenoidal rostrum process approach surgery was performed to remove the tumor (c shows the endoscopic image after sphenoid sinus septum resection [file HED-41-440-s001.zip › hed25435-sup-0003-FigureS3c.tif]

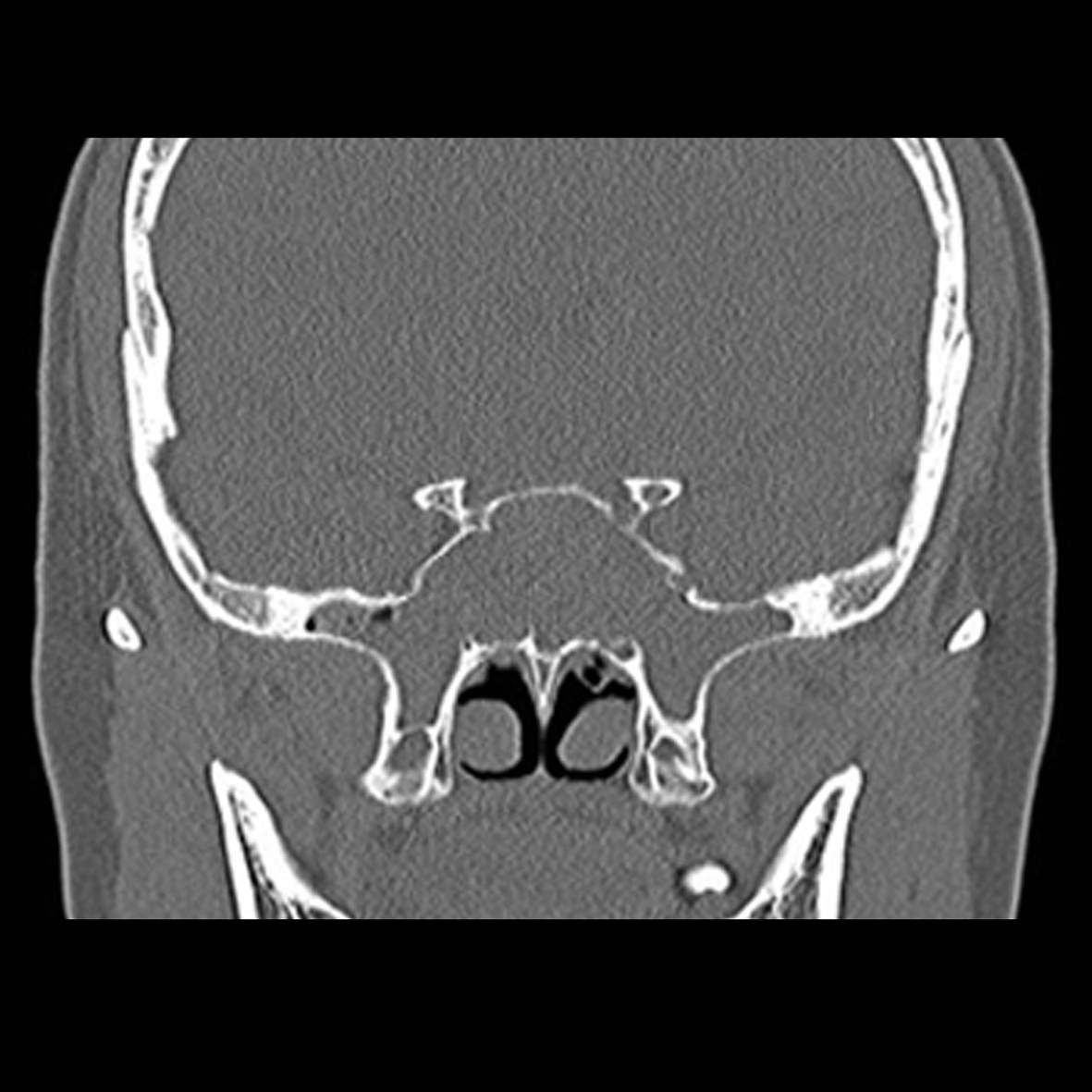

Supplement: Supplementary file 1 — Figure S1 Coronal contrast‐enhanced T1‐weighted MRI image (a) and nasal endoscopic examination (b) of a 45 years old woman, showing sinonasal inverted papilloma originating from left inferior turbinate (a, black circle). Endoscopic sinus surgery (ESS) was performed to remove the tumor, and 6 years after left inferior turbinate resection, there is no recurrence to date, as indicated by absence of tumor by CT scan (c) and endoscopy (d). Figure S2 Axial CT scan (a) and contrast‐enhanced T1‐weighted MRI image (b) of a 55 years old male, showing sinonasal inverted papilloma originating from posterior wall of left maxillary sinus (a, black circle; b, white circle). Endoscopic sinus surgery (ESS) was performed to remove the originating site of the tumor under 70° endoscopy (c ‐ tumor indicated by black arrow; d ‐ the tumor origin site after burning); with no recurrence to date 3 years after endoscopic sinus surgery. Figure S3 Axial (a) and coronal (b) contrast‐enhanced T1‐weighted MRI image of a 49 years old male, showing sinonasal inverted papilloma originating from anterior wall of left maxillary sinus (white circles). Endoscopic sinus surgery (ESS)‐assisted prelacrimal duct approach surgery was performed to remove the tumor (c ‐ black arrow indicates the originating site of the tumor, the white dotted lines indicated the lacrimal duct). Suppl. Figure 3d shows the origin site under 70° endoscopy after tumor resection (white dotted circle), A, I, M represented anterior, inferior, and medial wall of the maxillary sinus respectively; with no tumor recurrence after 5 years' follow‐up. Figure S4 Coronal CT scan (a) and contrast‐enhanced T1‐weighted MRI image (b) of a 59 years old female, showing sinonasal inverted papilloma originating from sphenoid sinus septum and affecting bilateral sinus. Endoscopic sinus surgery (ESS)‐assisted sphenoidal rostrum process approach surgery was performed to remove the tumor (c shows the endoscopic image after sphenoid sinus septum resection [file HED-41-440-s001.zip › hed25435-sup-0004-FigureS4.tif]

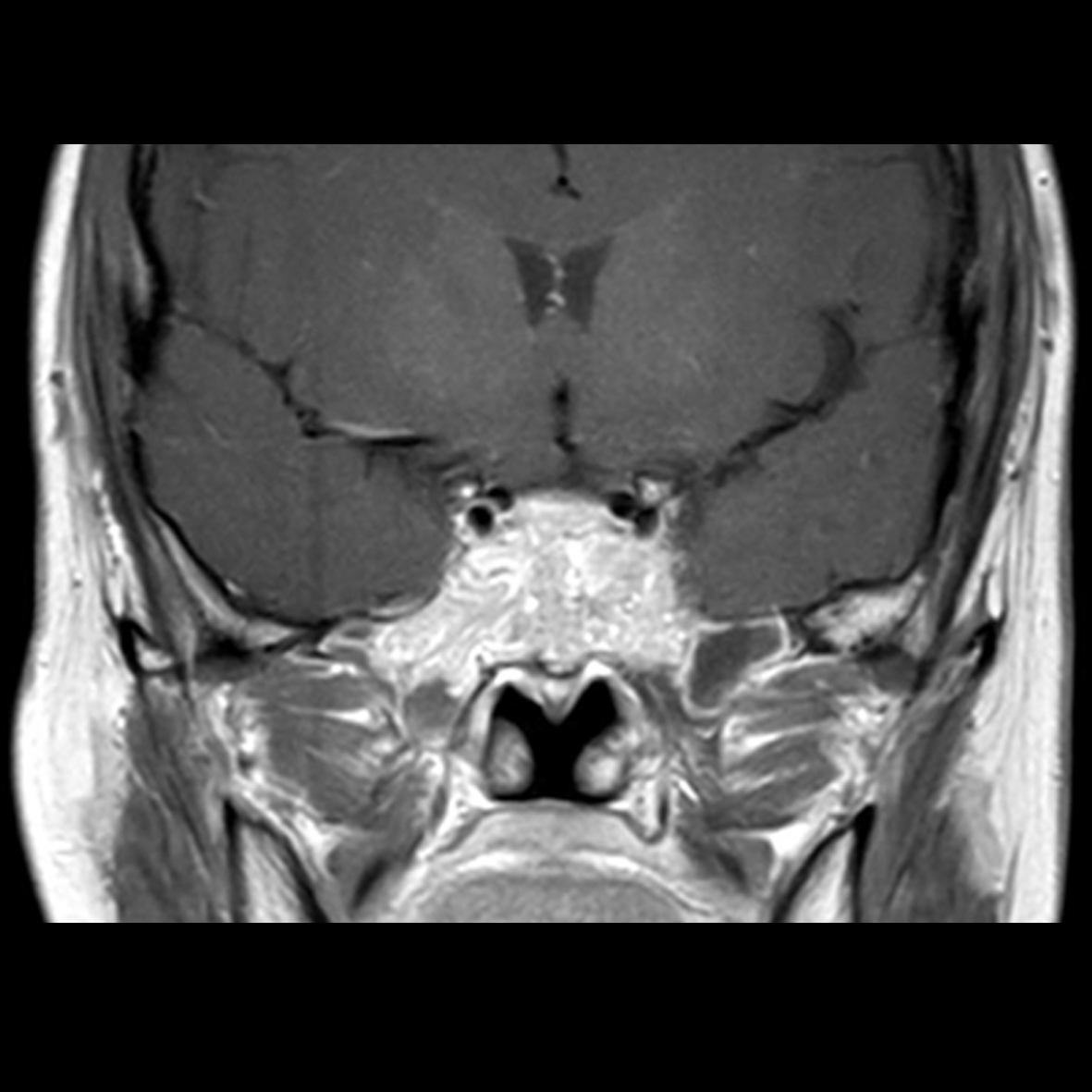

Supplement: Supplementary file 1 — Figure S1 Coronal contrast‐enhanced T1‐weighted MRI image (a) and nasal endoscopic examination (b) of a 45 years old woman, showing sinonasal inverted papilloma originating from left inferior turbinate (a, black circle). Endoscopic sinus surgery (ESS) was performed to remove the tumor, and 6 years after left inferior turbinate resection, there is no recurrence to date, as indicated by absence of tumor by CT scan (c) and endoscopy (d). Figure S2 Axial CT scan (a) and contrast‐enhanced T1‐weighted MRI image (b) of a 55 years old male, showing sinonasal inverted papilloma originating from posterior wall of left maxillary sinus (a, black circle; b, white circle). Endoscopic sinus surgery (ESS) was performed to remove the originating site of the tumor under 70° endoscopy (c ‐ tumor indicated by black arrow; d ‐ the tumor origin site after burning); with no recurrence to date 3 years after endoscopic sinus surgery. Figure S3 Axial (a) and coronal (b) contrast‐enhanced T1‐weighted MRI image of a 49 years old male, showing sinonasal inverted papilloma originating from anterior wall of left maxillary sinus (white circles). Endoscopic sinus surgery (ESS)‐assisted prelacrimal duct approach surgery was performed to remove the tumor (c ‐ black arrow indicates the originating site of the tumor, the white dotted lines indicated the lacrimal duct). Suppl. Figure 3d shows the origin site under 70° endoscopy after tumor resection (white dotted circle), A, I, M represented anterior, inferior, and medial wall of the maxillary sinus respectively; with no tumor recurrence after 5 years' follow‐up. Figure S4 Coronal CT scan (a) and contrast‐enhanced T1‐weighted MRI image (b) of a 59 years old female, showing sinonasal inverted papilloma originating from sphenoid sinus septum and affecting bilateral sinus. Endoscopic sinus surgery (ESS)‐assisted sphenoidal rostrum process approach surgery was performed to remove the tumor (c shows the endoscopic image after sphenoid sinus septum resection [file HED-41-440-s001.zip › hed25435-sup-0004-FigureS4a.tif]

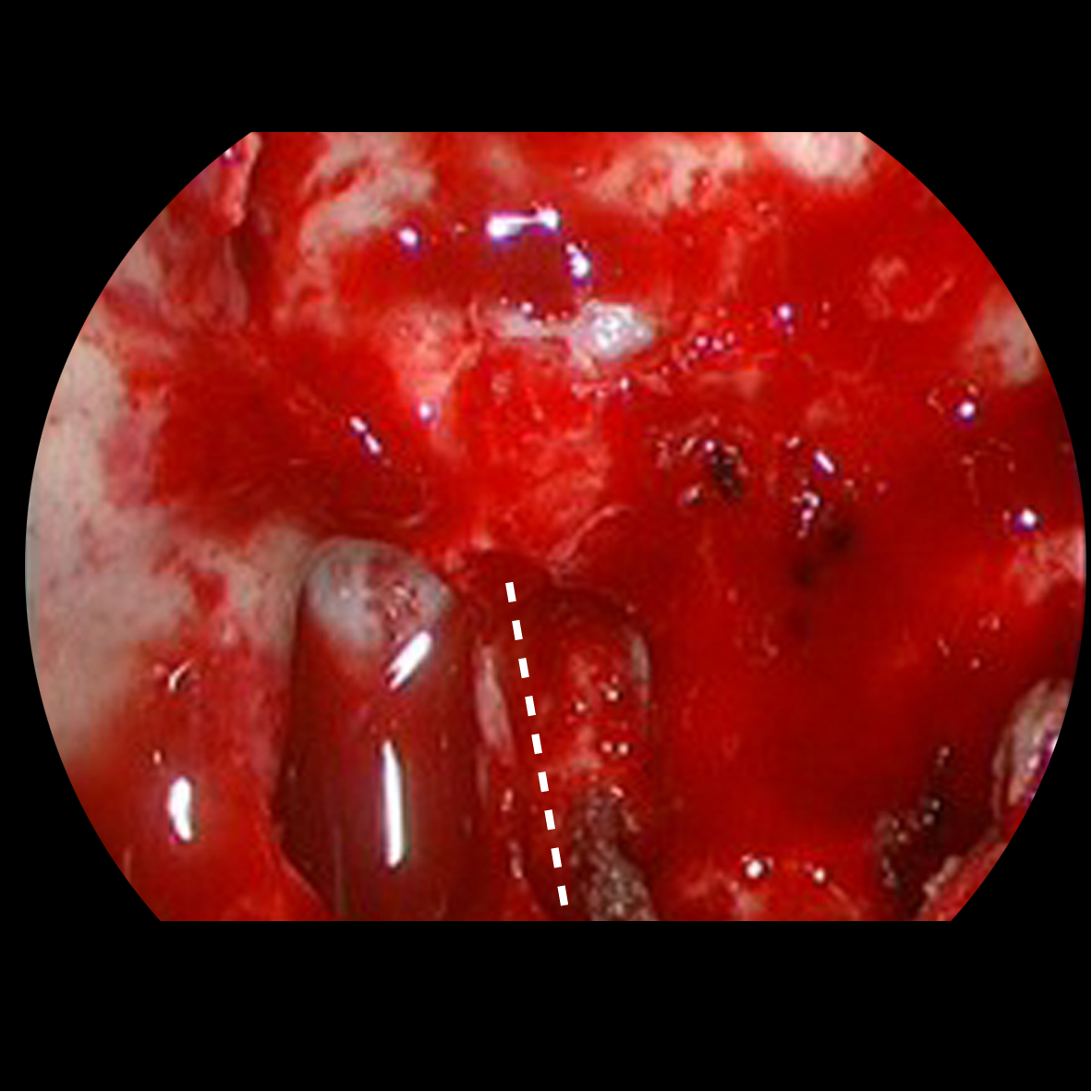

Supplement: Supplementary file 1 — Figure S1 Coronal contrast‐enhanced T1‐weighted MRI image (a) and nasal endoscopic examination (b) of a 45 years old woman, showing sinonasal inverted papilloma originating from left inferior turbinate (a, black circle). Endoscopic sinus surgery (ESS) was performed to remove the tumor, and 6 years after left inferior turbinate resection, there is no recurrence to date, as indicated by absence of tumor by CT scan (c) and endoscopy (d). Figure S2 Axial CT scan (a) and contrast‐enhanced T1‐weighted MRI image (b) of a 55 years old male, showing sinonasal inverted papilloma originating from posterior wall of left maxillary sinus (a, black circle; b, white circle). Endoscopic sinus surgery (ESS) was performed to remove the originating site of the tumor under 70° endoscopy (c ‐ tumor indicated by black arrow; d ‐ the tumor origin site after burning); with no recurrence to date 3 years after endoscopic sinus surgery. Figure S3 Axial (a) and coronal (b) contrast‐enhanced T1‐weighted MRI image of a 49 years old male, showing sinonasal inverted papilloma originating from anterior wall of left maxillary sinus (white circles). Endoscopic sinus surgery (ESS)‐assisted prelacrimal duct approach surgery was performed to remove the tumor (c ‐ black arrow indicates the originating site of the tumor, the white dotted lines indicated the lacrimal duct). Suppl. Figure 3d shows the origin site under 70° endoscopy after tumor resection (white dotted circle), A, I, M represented anterior, inferior, and medial wall of the maxillary sinus respectively; with no tumor recurrence after 5 years' follow‐up. Figure S4 Coronal CT scan (a) and contrast‐enhanced T1‐weighted MRI image (b) of a 59 years old female, showing sinonasal inverted papilloma originating from sphenoid sinus septum and affecting bilateral sinus. Endoscopic sinus surgery (ESS)‐assisted sphenoidal rostrum process approach surgery was performed to remove the tumor (c shows the endoscopic image after sphenoid sinus septum resection [file HED-41-440-s001.zip › hed25435-sup-0004-FigureS4b.tif]

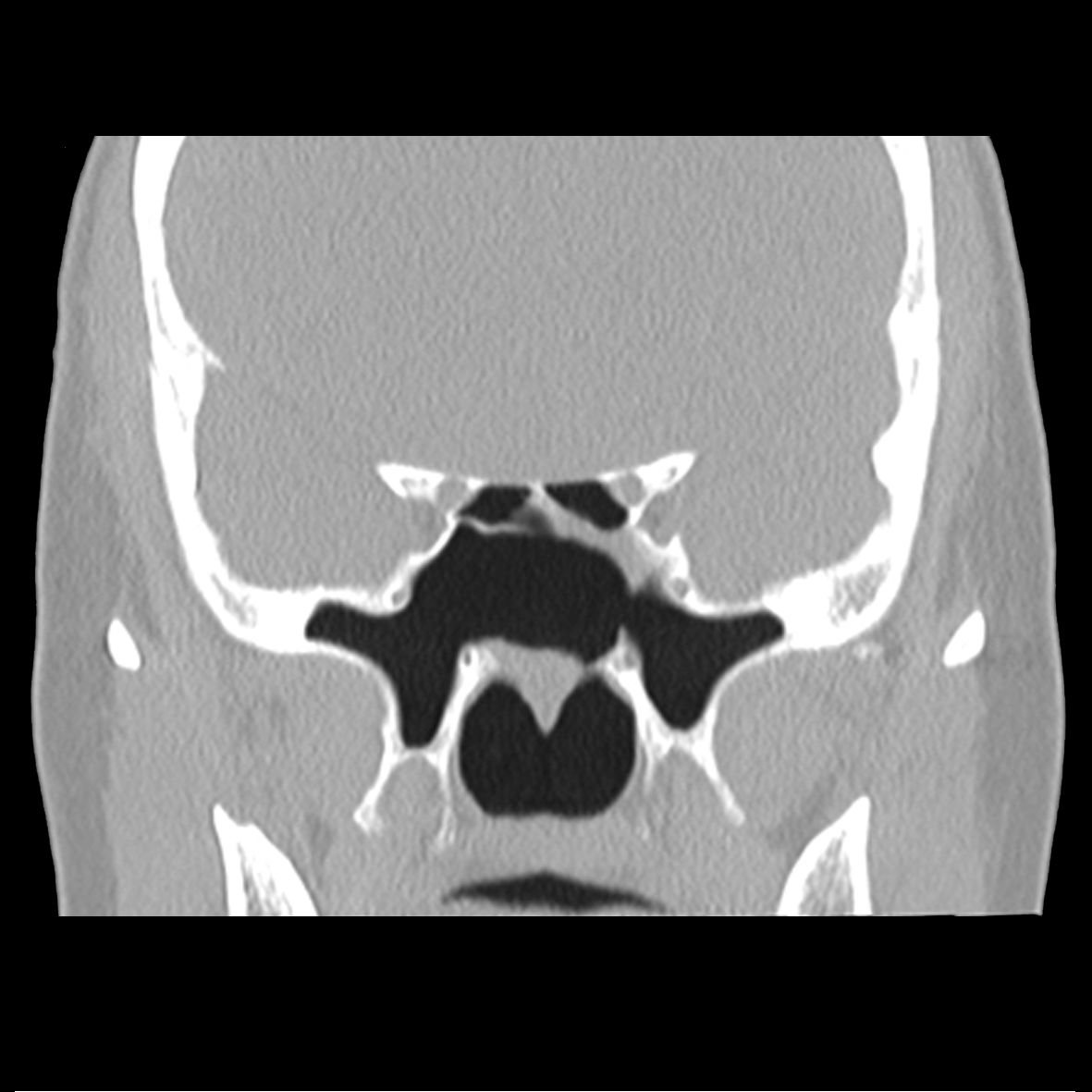

Supplement: Supplementary file 1 — Figure S1 Coronal contrast‐enhanced T1‐weighted MRI image (a) and nasal endoscopic examination (b) of a 45 years old woman, showing sinonasal inverted papilloma originating from left inferior turbinate (a, black circle). Endoscopic sinus surgery (ESS) was performed to remove the tumor, and 6 years after left inferior turbinate resection, there is no recurrence to date, as indicated by absence of tumor by CT scan (c) and endoscopy (d). Figure S2 Axial CT scan (a) and contrast‐enhanced T1‐weighted MRI image (b) of a 55 years old male, showing sinonasal inverted papilloma originating from posterior wall of left maxillary sinus (a, black circle; b, white circle). Endoscopic sinus surgery (ESS) was performed to remove the originating site of the tumor under 70° endoscopy (c ‐ tumor indicated by black arrow; d ‐ the tumor origin site after burning); with no recurrence to date 3 years after endoscopic sinus surgery. Figure S3 Axial (a) and coronal (b) contrast‐enhanced T1‐weighted MRI image of a 49 years old male, showing sinonasal inverted papilloma originating from anterior wall of left maxillary sinus (white circles). Endoscopic sinus surgery (ESS)‐assisted prelacrimal duct approach surgery was performed to remove the tumor (c ‐ black arrow indicates the originating site of the tumor, the white dotted lines indicated the lacrimal duct). Suppl. Figure 3d shows the origin site under 70° endoscopy after tumor resection (white dotted circle), A, I, M represented anterior, inferior, and medial wall of the maxillary sinus respectively; with no tumor recurrence after 5 years' follow‐up. Figure S4 Coronal CT scan (a) and contrast‐enhanced T1‐weighted MRI image (b) of a 59 years old female, showing sinonasal inverted papilloma originating from sphenoid sinus septum and affecting bilateral sinus. Endoscopic sinus surgery (ESS)‐assisted sphenoidal rostrum process approach surgery was performed to remove the tumor (c shows the endoscopic image after sphenoid sinus septum resection [file HED-41-440-s001.zip › hed25435-sup-0004-FigureS4c.tif]

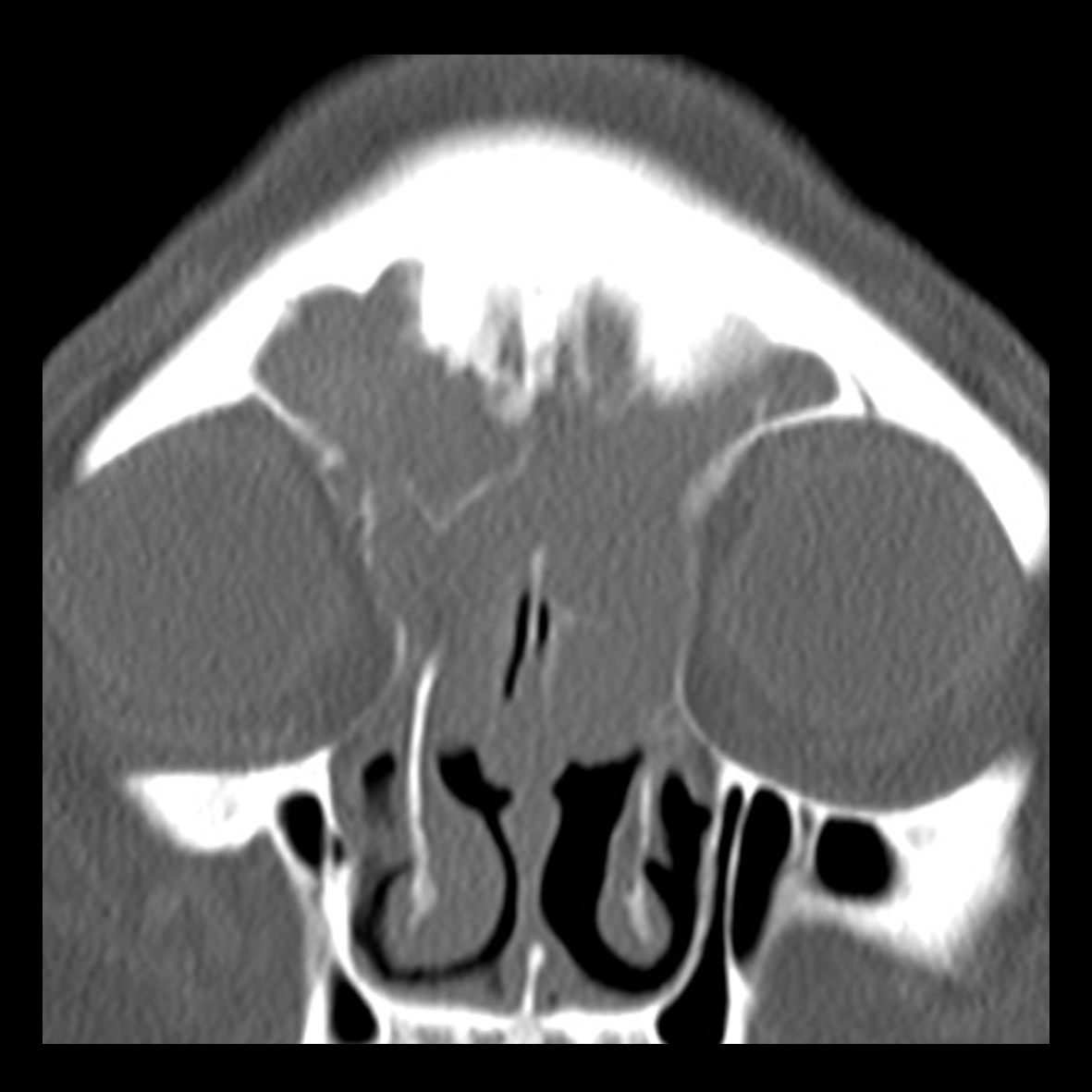

Supplement: Supplementary file 1 — Figure S1 Coronal contrast‐enhanced T1‐weighted MRI image (a) and nasal endoscopic examination (b) of a 45 years old woman, showing sinonasal inverted papilloma originating from left inferior turbinate (a, black circle). Endoscopic sinus surgery (ESS) was performed to remove the tumor, and 6 years after left inferior turbinate resection, there is no recurrence to date, as indicated by absence of tumor by CT scan (c) and endoscopy (d). Figure S2 Axial CT scan (a) and contrast‐enhanced T1‐weighted MRI image (b) of a 55 years old male, showing sinonasal inverted papilloma originating from posterior wall of left maxillary sinus (a, black circle; b, white circle). Endoscopic sinus surgery (ESS) was performed to remove the originating site of the tumor under 70° endoscopy (c ‐ tumor indicated by black arrow; d ‐ the tumor origin site after burning); with no recurrence to date 3 years after endoscopic sinus surgery. Figure S3 Axial (a) and coronal (b) contrast‐enhanced T1‐weighted MRI image of a 49 years old male, showing sinonasal inverted papilloma originating from anterior wall of left maxillary sinus (white circles). Endoscopic sinus surgery (ESS)‐assisted prelacrimal duct approach surgery was performed to remove the tumor (c ‐ black arrow indicates the originating site of the tumor, the white dotted lines indicated the lacrimal duct). Suppl. Figure 3d shows the origin site under 70° endoscopy after tumor resection (white dotted circle), A, I, M represented anterior, inferior, and medial wall of the maxillary sinus respectively; with no tumor recurrence after 5 years' follow‐up. Figure S4 Coronal CT scan (a) and contrast‐enhanced T1‐weighted MRI image (b) of a 59 years old female, showing sinonasal inverted papilloma originating from sphenoid sinus septum and affecting bilateral sinus. Endoscopic sinus surgery (ESS)‐assisted sphenoidal rostrum process approach surgery was performed to remove the tumor (c shows the endoscopic image after sphenoid sinus septum resection [file HED-41-440-s001.zip › hed25435-sup-0005-FigureS5.tif]

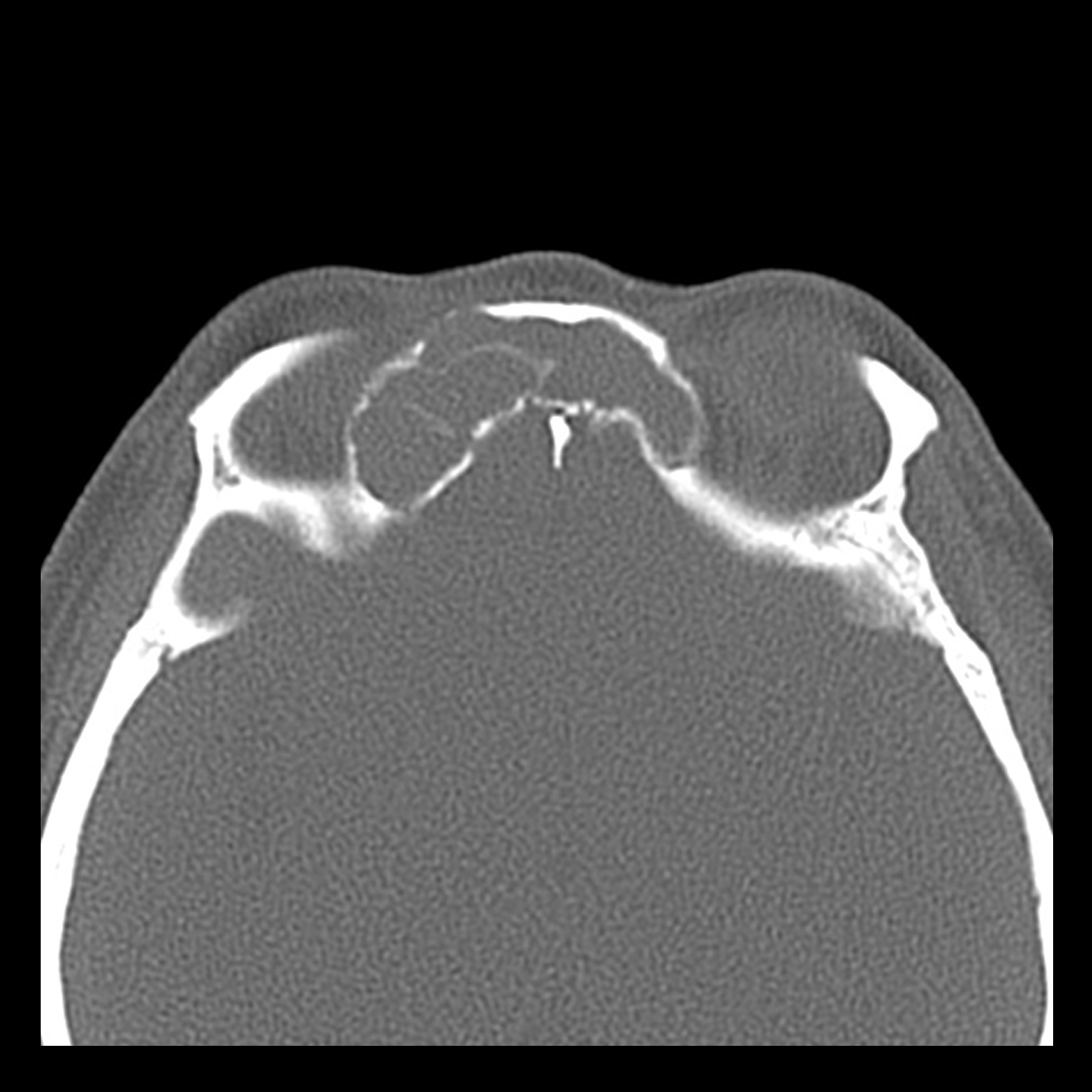

Supplement: Supplementary file 1 — Figure S1 Coronal contrast‐enhanced T1‐weighted MRI image (a) and nasal endoscopic examination (b) of a 45 years old woman, showing sinonasal inverted papilloma originating from left inferior turbinate (a, black circle). Endoscopic sinus surgery (ESS) was performed to remove the tumor, and 6 years after left inferior turbinate resection, there is no recurrence to date, as indicated by absence of tumor by CT scan (c) and endoscopy (d). Figure S2 Axial CT scan (a) and contrast‐enhanced T1‐weighted MRI image (b) of a 55 years old male, showing sinonasal inverted papilloma originating from posterior wall of left maxillary sinus (a, black circle; b, white circle). Endoscopic sinus surgery (ESS) was performed to remove the originating site of the tumor under 70° endoscopy (c ‐ tumor indicated by black arrow; d ‐ the tumor origin site after burning); with no recurrence to date 3 years after endoscopic sinus surgery. Figure S3 Axial (a) and coronal (b) contrast‐enhanced T1‐weighted MRI image of a 49 years old male, showing sinonasal inverted papilloma originating from anterior wall of left maxillary sinus (white circles). Endoscopic sinus surgery (ESS)‐assisted prelacrimal duct approach surgery was performed to remove the tumor (c ‐ black arrow indicates the originating site of the tumor, the white dotted lines indicated the lacrimal duct). Suppl. Figure 3d shows the origin site under 70° endoscopy after tumor resection (white dotted circle), A, I, M represented anterior, inferior, and medial wall of the maxillary sinus respectively; with no tumor recurrence after 5 years' follow‐up. Figure S4 Coronal CT scan (a) and contrast‐enhanced T1‐weighted MRI image (b) of a 59 years old female, showing sinonasal inverted papilloma originating from sphenoid sinus septum and affecting bilateral sinus. Endoscopic sinus surgery (ESS)‐assisted sphenoidal rostrum process approach surgery was performed to remove the tumor (c shows the endoscopic image after sphenoid sinus septum resection [file HED-41-440-s001.zip › hed25435-sup-0005-FigureS5a.tif]

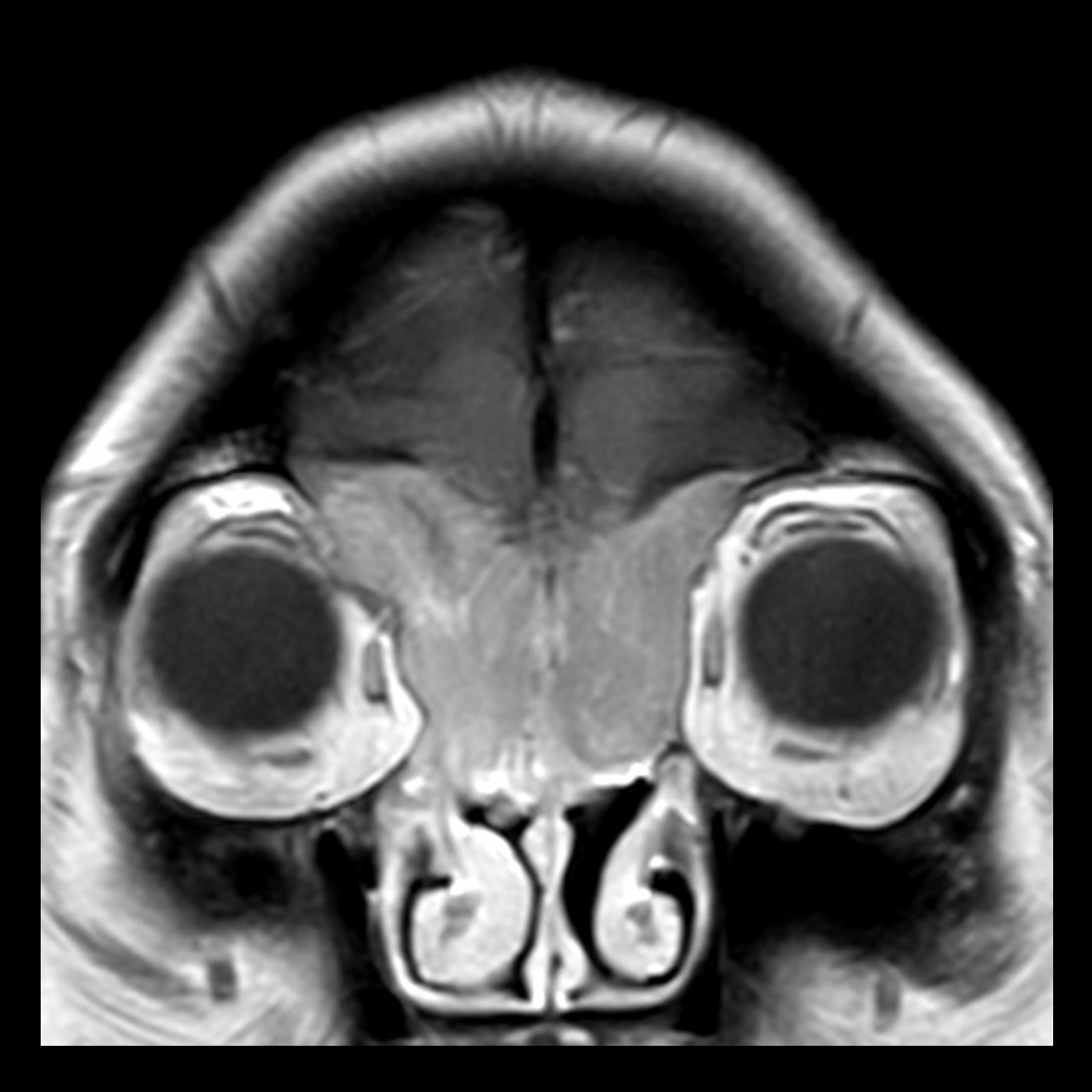

Supplement: Supplementary file 1 — Figure S1 Coronal contrast‐enhanced T1‐weighted MRI image (a) and nasal endoscopic examination (b) of a 45 years old woman, showing sinonasal inverted papilloma originating from left inferior turbinate (a, black circle). Endoscopic sinus surgery (ESS) was performed to remove the tumor, and 6 years after left inferior turbinate resection, there is no recurrence to date, as indicated by absence of tumor by CT scan (c) and endoscopy (d). Figure S2 Axial CT scan (a) and contrast‐enhanced T1‐weighted MRI image (b) of a 55 years old male, showing sinonasal inverted papilloma originating from posterior wall of left maxillary sinus (a, black circle; b, white circle). Endoscopic sinus surgery (ESS) was performed to remove the originating site of the tumor under 70° endoscopy (c ‐ tumor indicated by black arrow; d ‐ the tumor origin site after burning); with no recurrence to date 3 years after endoscopic sinus surgery. Figure S3 Axial (a) and coronal (b) contrast‐enhanced T1‐weighted MRI image of a 49 years old male, showing sinonasal inverted papilloma originating from anterior wall of left maxillary sinus (white circles). Endoscopic sinus surgery (ESS)‐assisted prelacrimal duct approach surgery was performed to remove the tumor (c ‐ black arrow indicates the originating site of the tumor, the white dotted lines indicated the lacrimal duct). Suppl. Figure 3d shows the origin site under 70° endoscopy after tumor resection (white dotted circle), A, I, M represented anterior, inferior, and medial wall of the maxillary sinus respectively; with no tumor recurrence after 5 years' follow‐up. Figure S4 Coronal CT scan (a) and contrast‐enhanced T1‐weighted MRI image (b) of a 59 years old female, showing sinonasal inverted papilloma originating from sphenoid sinus septum and affecting bilateral sinus. Endoscopic sinus surgery (ESS)‐assisted sphenoidal rostrum process approach surgery was performed to remove the tumor (c shows the endoscopic image after sphenoid sinus septum resection [file HED-41-440-s001.zip › hed25435-sup-0005-FigureS5b.tif]

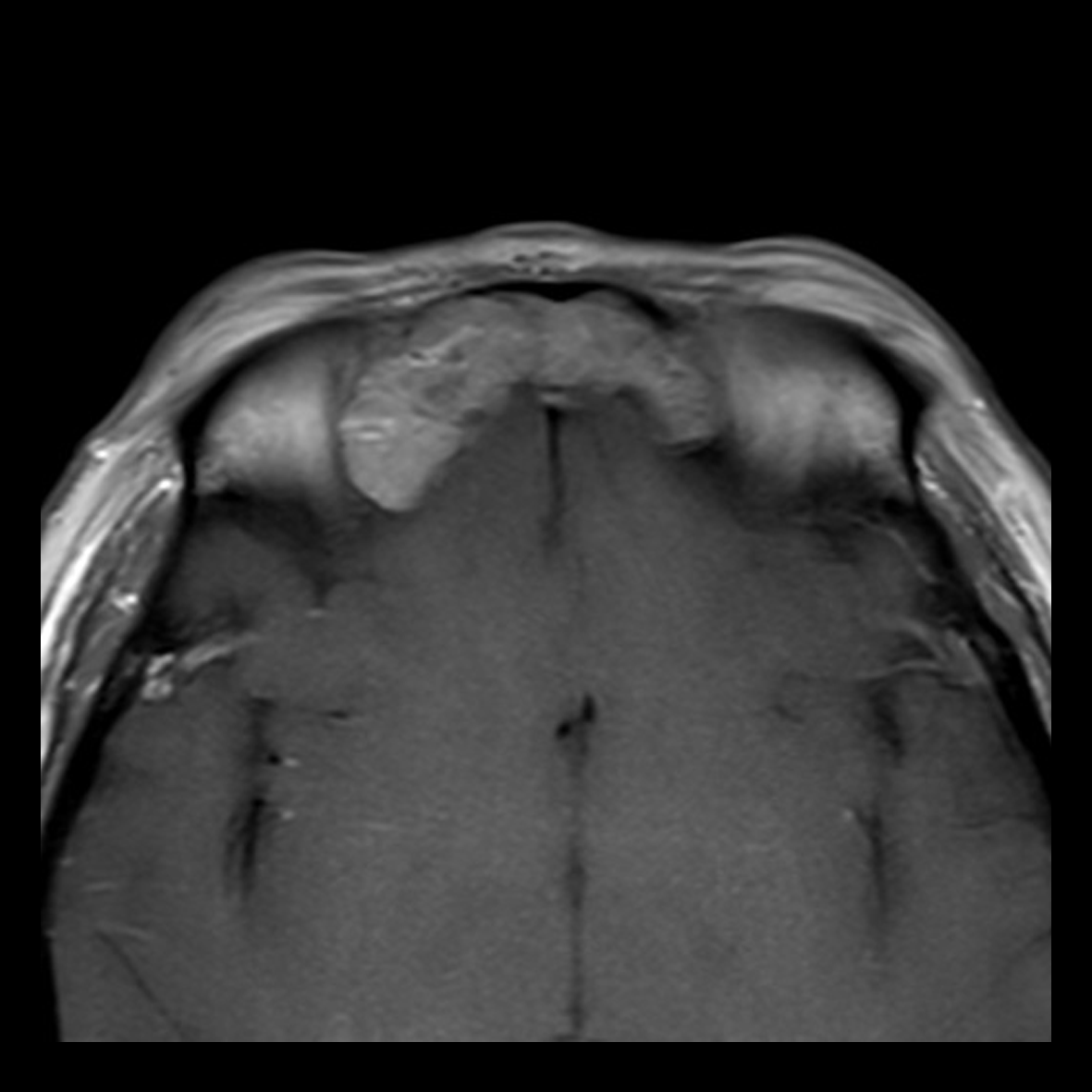

Supplement: Supplementary file 1 — Figure S1 Coronal contrast‐enhanced T1‐weighted MRI image (a) and nasal endoscopic examination (b) of a 45 years old woman, showing sinonasal inverted papilloma originating from left inferior turbinate (a, black circle). Endoscopic sinus surgery (ESS) was performed to remove the tumor, and 6 years after left inferior turbinate resection, there is no recurrence to date, as indicated by absence of tumor by CT scan (c) and endoscopy (d). Figure S2 Axial CT scan (a) and contrast‐enhanced T1‐weighted MRI image (b) of a 55 years old male, showing sinonasal inverted papilloma originating from posterior wall of left maxillary sinus (a, black circle; b, white circle). Endoscopic sinus surgery (ESS) was performed to remove the originating site of the tumor under 70° endoscopy (c ‐ tumor indicated by black arrow; d ‐ the tumor origin site after burning); with no recurrence to date 3 years after endoscopic sinus surgery. Figure S3 Axial (a) and coronal (b) contrast‐enhanced T1‐weighted MRI image of a 49 years old male, showing sinonasal inverted papilloma originating from anterior wall of left maxillary sinus (white circles). Endoscopic sinus surgery (ESS)‐assisted prelacrimal duct approach surgery was performed to remove the tumor (c ‐ black arrow indicates the originating site of the tumor, the white dotted lines indicated the lacrimal duct). Suppl. Figure 3d shows the origin site under 70° endoscopy after tumor resection (white dotted circle), A, I, M represented anterior, inferior, and medial wall of the maxillary sinus respectively; with no tumor recurrence after 5 years' follow‐up. Figure S4 Coronal CT scan (a) and contrast‐enhanced T1‐weighted MRI image (b) of a 59 years old female, showing sinonasal inverted papilloma originating from sphenoid sinus septum and affecting bilateral sinus. Endoscopic sinus surgery (ESS)‐assisted sphenoidal rostrum process approach surgery was performed to remove the tumor (c shows the endoscopic image after sphenoid sinus septum resection [file HED-41-440-s001.zip › hed25435-sup-0005-FigureS5c.tif]

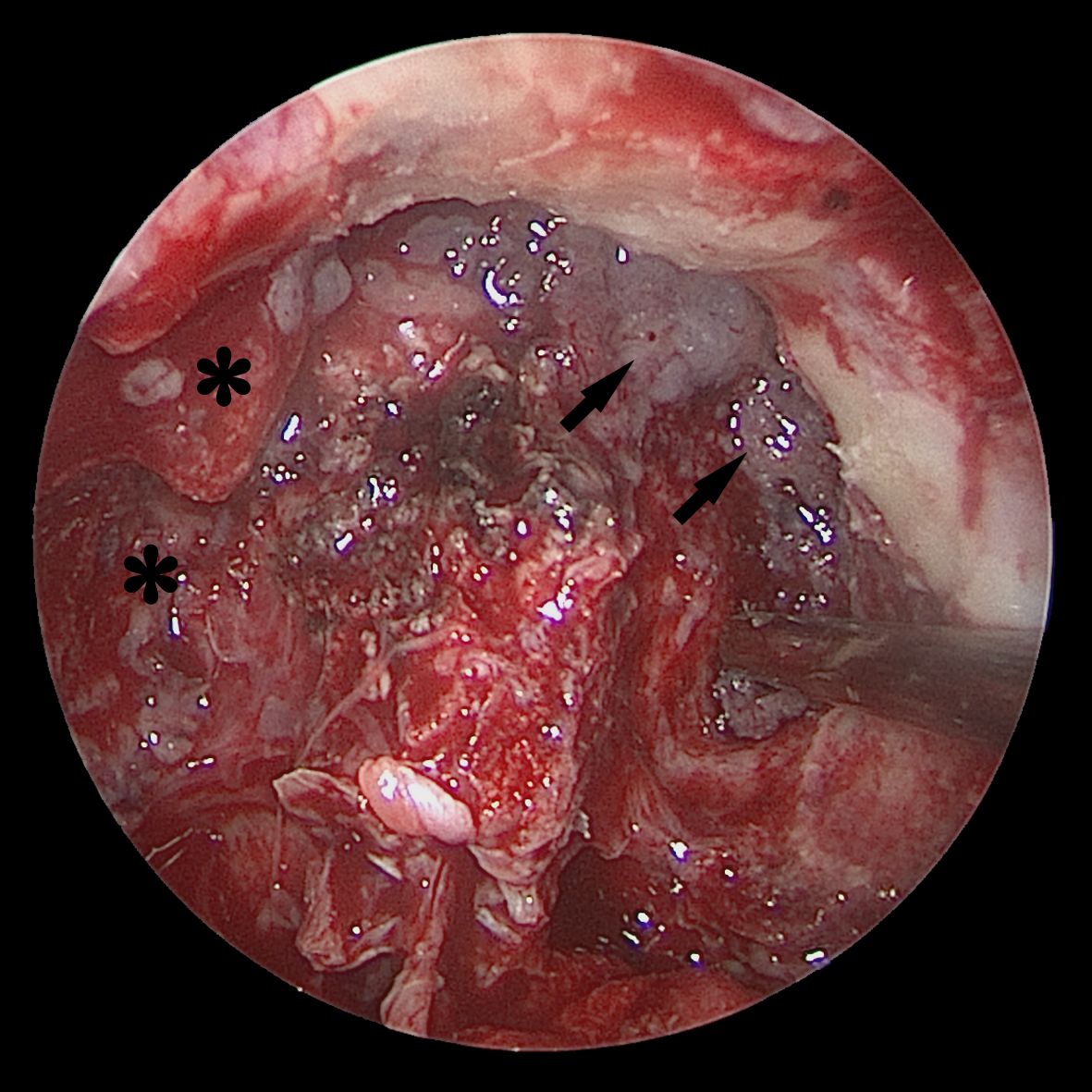

Supplement: Supplementary file 1 — Figure S1 Coronal contrast‐enhanced T1‐weighted MRI image (a) and nasal endoscopic examination (b) of a 45 years old woman, showing sinonasal inverted papilloma originating from left inferior turbinate (a, black circle). Endoscopic sinus surgery (ESS) was performed to remove the tumor, and 6 years after left inferior turbinate resection, there is no recurrence to date, as indicated by absence of tumor by CT scan (c) and endoscopy (d). Figure S2 Axial CT scan (a) and contrast‐enhanced T1‐weighted MRI image (b) of a 55 years old male, showing sinonasal inverted papilloma originating from posterior wall of left maxillary sinus (a, black circle; b, white circle). Endoscopic sinus surgery (ESS) was performed to remove the originating site of the tumor under 70° endoscopy (c ‐ tumor indicated by black arrow; d ‐ the tumor origin site after burning); with no recurrence to date 3 years after endoscopic sinus surgery. Figure S3 Axial (a) and coronal (b) contrast‐enhanced T1‐weighted MRI image of a 49 years old male, showing sinonasal inverted papilloma originating from anterior wall of left maxillary sinus (white circles). Endoscopic sinus surgery (ESS)‐assisted prelacrimal duct approach surgery was performed to remove the tumor (c ‐ black arrow indicates the originating site of the tumor, the white dotted lines indicated the lacrimal duct). Suppl. Figure 3d shows the origin site under 70° endoscopy after tumor resection (white dotted circle), A, I, M represented anterior, inferior, and medial wall of the maxillary sinus respectively; with no tumor recurrence after 5 years' follow‐up. Figure S4 Coronal CT scan (a) and contrast‐enhanced T1‐weighted MRI image (b) of a 59 years old female, showing sinonasal inverted papilloma originating from sphenoid sinus septum and affecting bilateral sinus. Endoscopic sinus surgery (ESS)‐assisted sphenoidal rostrum process approach surgery was performed to remove the tumor (c shows the endoscopic image after sphenoid sinus septum resection [file HED-41-440-s001.zip › hed25435-sup-0005-FigureS5d.tif]

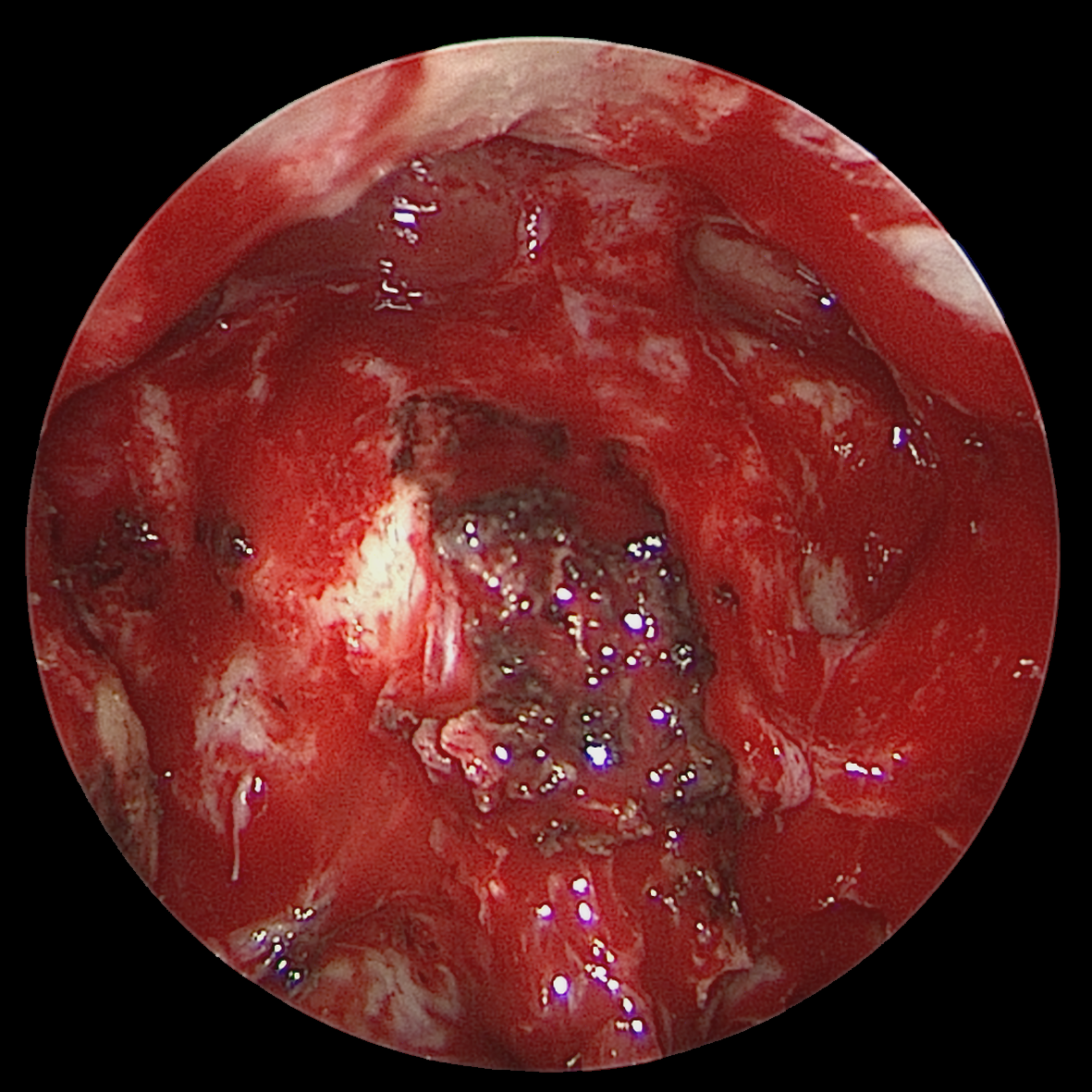

Supplement: Supplementary file 1 — Figure S1 Coronal contrast‐enhanced T1‐weighted MRI image (a) and nasal endoscopic examination (b) of a 45 years old woman, showing sinonasal inverted papilloma originating from left inferior turbinate (a, black circle). Endoscopic sinus surgery (ESS) was performed to remove the tumor, and 6 years after left inferior turbinate resection, there is no recurrence to date, as indicated by absence of tumor by CT scan (c) and endoscopy (d). Figure S2 Axial CT scan (a) and contrast‐enhanced T1‐weighted MRI image (b) of a 55 years old male, showing sinonasal inverted papilloma originating from posterior wall of left maxillary sinus (a, black circle; b, white circle). Endoscopic sinus surgery (ESS) was performed to remove the originating site of the tumor under 70° endoscopy (c ‐ tumor indicated by black arrow; d ‐ the tumor origin site after burning); with no recurrence to date 3 years after endoscopic sinus surgery. Figure S3 Axial (a) and coronal (b) contrast‐enhanced T1‐weighted MRI image of a 49 years old male, showing sinonasal inverted papilloma originating from anterior wall of left maxillary sinus (white circles). Endoscopic sinus surgery (ESS)‐assisted prelacrimal duct approach surgery was performed to remove the tumor (c ‐ black arrow indicates the originating site of the tumor, the white dotted lines indicated the lacrimal duct). Suppl. Figure 3d shows the origin site under 70° endoscopy after tumor resection (white dotted circle), A, I, M represented anterior, inferior, and medial wall of the maxillary sinus respectively; with no tumor recurrence after 5 years' follow‐up. Figure S4 Coronal CT scan (a) and contrast‐enhanced T1‐weighted MRI image (b) of a 59 years old female, showing sinonasal inverted papilloma originating from sphenoid sinus septum and affecting bilateral sinus. Endoscopic sinus surgery (ESS)‐assisted sphenoidal rostrum process approach surgery was performed to remove the tumor (c shows the endoscopic image after sphenoid sinus septum resection [file HED-41-440-s001.zip › hed25435-sup-0005-FigureS5e.tif]

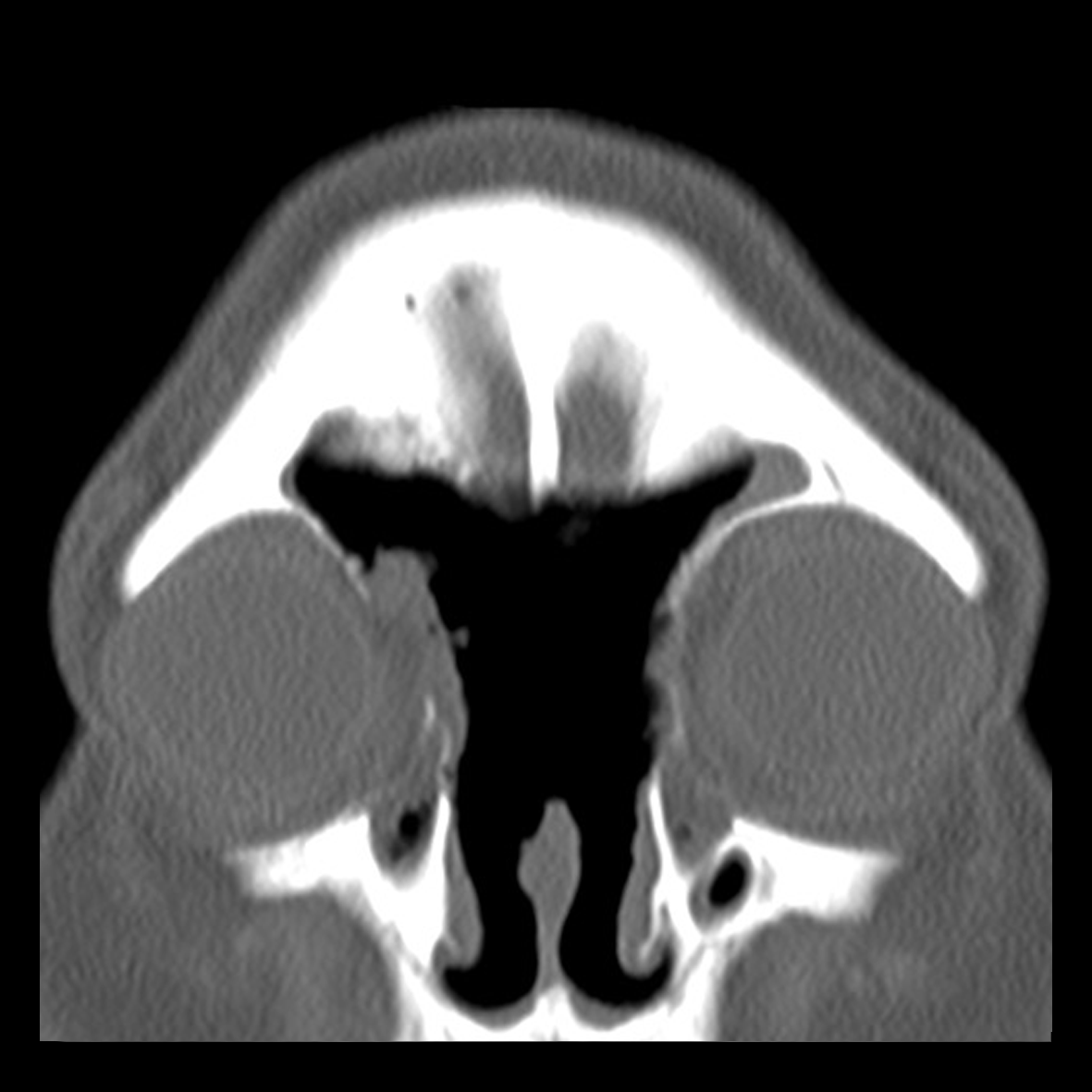

Supplement: Supplementary file 1 — Figure S1 Coronal contrast‐enhanced T1‐weighted MRI image (a) and nasal endoscopic examination (b) of a 45 years old woman, showing sinonasal inverted papilloma originating from left inferior turbinate (a, black circle). Endoscopic sinus surgery (ESS) was performed to remove the tumor, and 6 years after left inferior turbinate resection, there is no recurrence to date, as indicated by absence of tumor by CT scan (c) and endoscopy (d). Figure S2 Axial CT scan (a) and contrast‐enhanced T1‐weighted MRI image (b) of a 55 years old male, showing sinonasal inverted papilloma originating from posterior wall of left maxillary sinus (a, black circle; b, white circle). Endoscopic sinus surgery (ESS) was performed to remove the originating site of the tumor under 70° endoscopy (c ‐ tumor indicated by black arrow; d ‐ the tumor origin site after burning); with no recurrence to date 3 years after endoscopic sinus surgery. Figure S3 Axial (a) and coronal (b) contrast‐enhanced T1‐weighted MRI image of a 49 years old male, showing sinonasal inverted papilloma originating from anterior wall of left maxillary sinus (white circles). Endoscopic sinus surgery (ESS)‐assisted prelacrimal duct approach surgery was performed to remove the tumor (c ‐ black arrow indicates the originating site of the tumor, the white dotted lines indicated the lacrimal duct). Suppl. Figure 3d shows the origin site under 70° endoscopy after tumor resection (white dotted circle), A, I, M represented anterior, inferior, and medial wall of the maxillary sinus respectively; with no tumor recurrence after 5 years' follow‐up. Figure S4 Coronal CT scan (a) and contrast‐enhanced T1‐weighted MRI image (b) of a 59 years old female, showing sinonasal inverted papilloma originating from sphenoid sinus septum and affecting bilateral sinus. Endoscopic sinus surgery (ESS)‐assisted sphenoidal rostrum process approach surgery was performed to remove the tumor (c shows the endoscopic image after sphenoid sinus septum resection [file HED-41-440-s001.zip › hed25435-sup-0005-FigureS5f.tif]

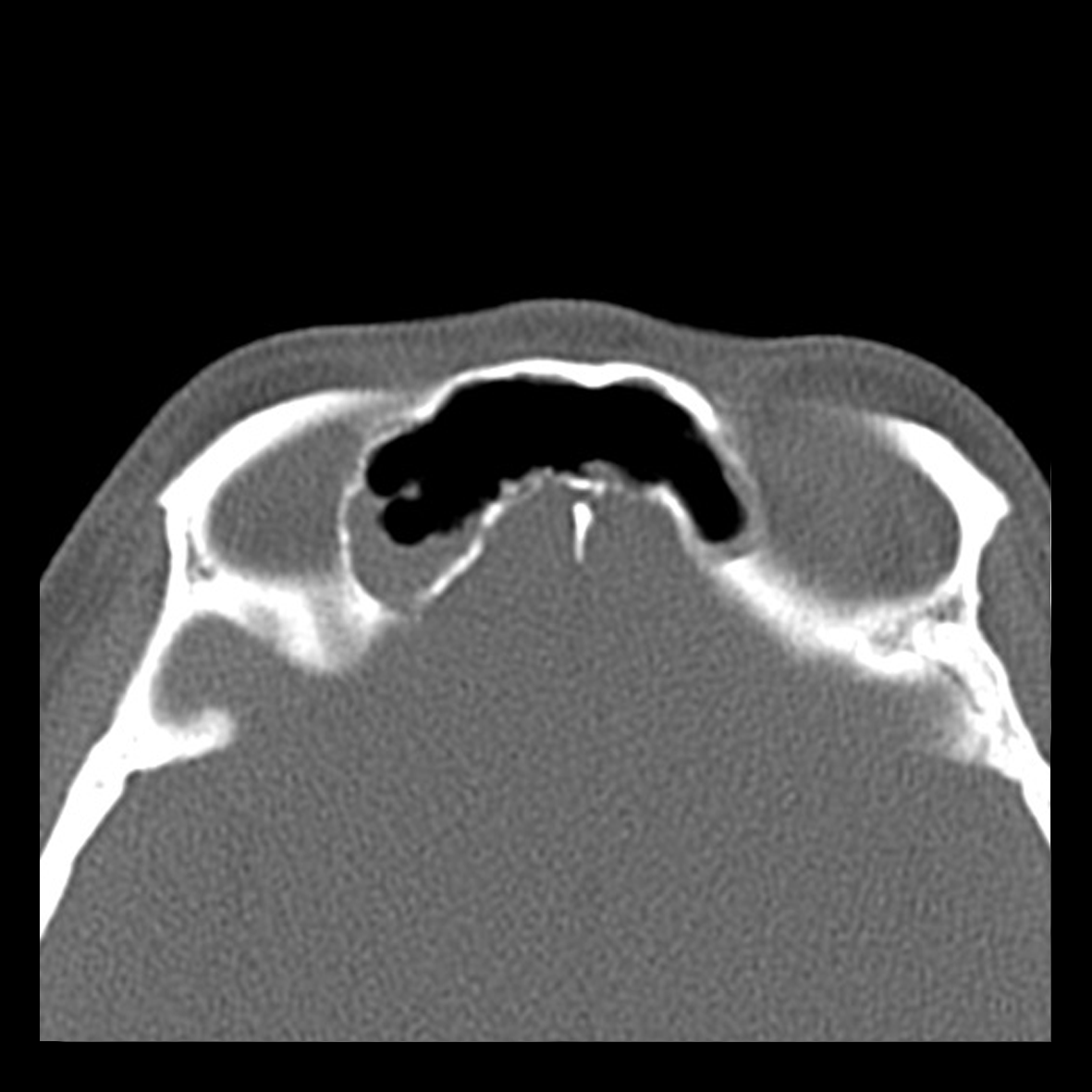

Supplement: Supplementary file 1 — Figure S1 Coronal contrast‐enhanced T1‐weighted MRI image (a) and nasal endoscopic examination (b) of a 45 years old woman, showing sinonasal inverted papilloma originating from left inferior turbinate (a, black circle). Endoscopic sinus surgery (ESS) was performed to remove the tumor, and 6 years after left inferior turbinate resection, there is no recurrence to date, as indicated by absence of tumor by CT scan (c) and endoscopy (d). Figure S2 Axial CT scan (a) and contrast‐enhanced T1‐weighted MRI image (b) of a 55 years old male, showing sinonasal inverted papilloma originating from posterior wall of left maxillary sinus (a, black circle; b, white circle). Endoscopic sinus surgery (ESS) was performed to remove the originating site of the tumor under 70° endoscopy (c ‐ tumor indicated by black arrow; d ‐ the tumor origin site after burning); with no recurrence to date 3 years after endoscopic sinus surgery. Figure S3 Axial (a) and coronal (b) contrast‐enhanced T1‐weighted MRI image of a 49 years old male, showing sinonasal inverted papilloma originating from anterior wall of left maxillary sinus (white circles). Endoscopic sinus surgery (ESS)‐assisted prelacrimal duct approach surgery was performed to remove the tumor (c ‐ black arrow indicates the originating site of the tumor, the white dotted lines indicated the lacrimal duct). Suppl. Figure 3d shows the origin site under 70° endoscopy after tumor resection (white dotted circle), A, I, M represented anterior, inferior, and medial wall of the maxillary sinus respectively; with no tumor recurrence after 5 years' follow‐up. Figure S4 Coronal CT scan (a) and contrast‐enhanced T1‐weighted MRI image (b) of a 59 years old female, showing sinonasal inverted papilloma originating from sphenoid sinus septum and affecting bilateral sinus. Endoscopic sinus surgery (ESS)‐assisted sphenoidal rostrum process approach surgery was performed to remove the tumor (c shows the endoscopic image after sphenoid sinus septum resection [file HED-41-440-s001.zip › hed25435-sup-0005-FigureS5g.tif]

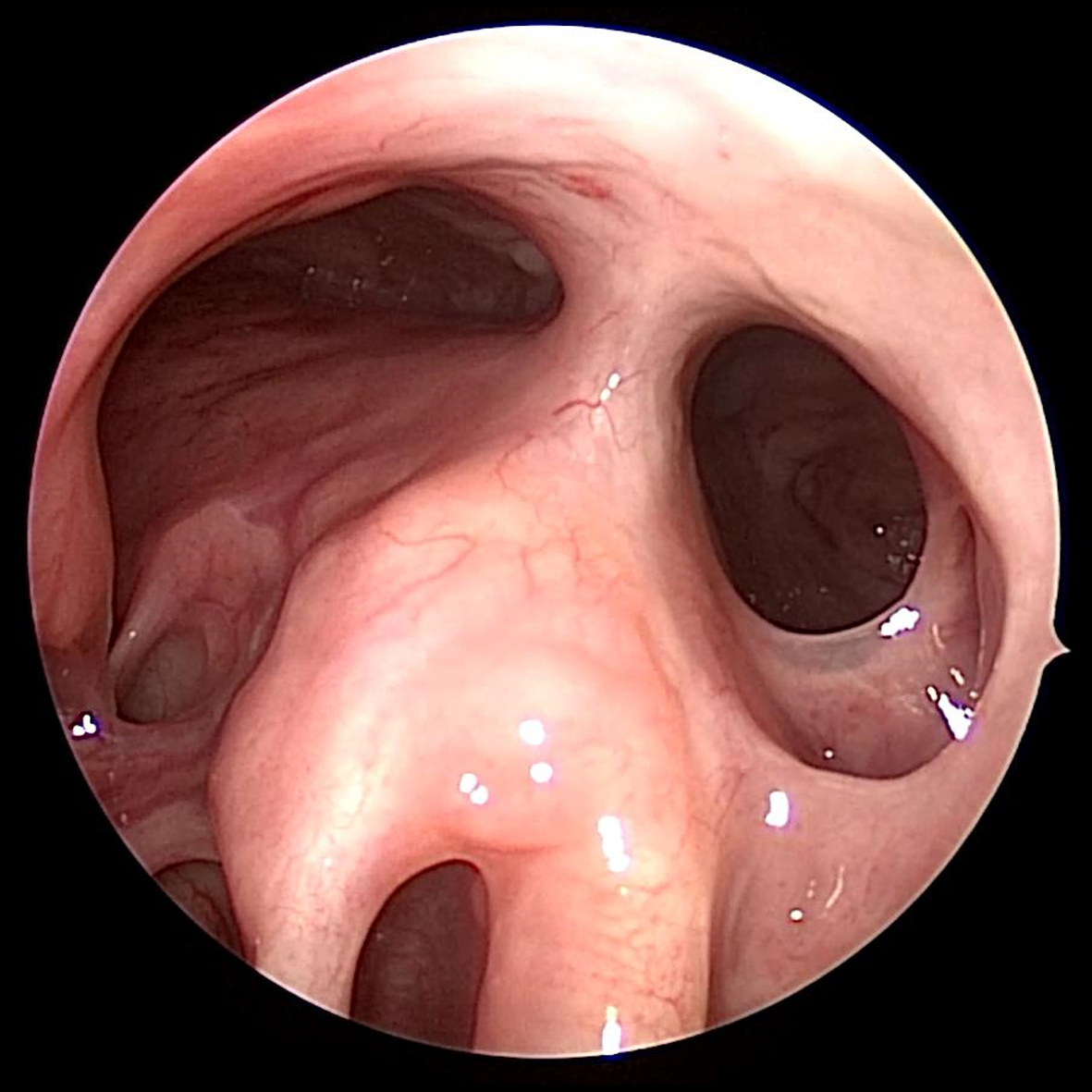

Supplement: Supplementary file 1 — Figure S1 Coronal contrast‐enhanced T1‐weighted MRI image (a) and nasal endoscopic examination (b) of a 45 years old woman, showing sinonasal inverted papilloma originating from left inferior turbinate (a, black circle). Endoscopic sinus surgery (ESS) was performed to remove the tumor, and 6 years after left inferior turbinate resection, there is no recurrence to date, as indicated by absence of tumor by CT scan (c) and endoscopy (d). Figure S2 Axial CT scan (a) and contrast‐enhanced T1‐weighted MRI image (b) of a 55 years old male, showing sinonasal inverted papilloma originating from posterior wall of left maxillary sinus (a, black circle; b, white circle). Endoscopic sinus surgery (ESS) was performed to remove the originating site of the tumor under 70° endoscopy (c ‐ tumor indicated by black arrow; d ‐ the tumor origin site after burning); with no recurrence to date 3 years after endoscopic sinus surgery. Figure S3 Axial (a) and coronal (b) contrast‐enhanced T1‐weighted MRI image of a 49 years old male, showing sinonasal inverted papilloma originating from anterior wall of left maxillary sinus (white circles). Endoscopic sinus surgery (ESS)‐assisted prelacrimal duct approach surgery was performed to remove the tumor (c ‐ black arrow indicates the originating site of the tumor, the white dotted lines indicated the lacrimal duct). Suppl. Figure 3d shows the origin site under 70° endoscopy after tumor resection (white dotted circle), A, I, M represented anterior, inferior, and medial wall of the maxillary sinus respectively; with no tumor recurrence after 5 years' follow‐up. Figure S4 Coronal CT scan (a) and contrast‐enhanced T1‐weighted MRI image (b) of a 59 years old female, showing sinonasal inverted papilloma originating from sphenoid sinus septum and affecting bilateral sinus. Endoscopic sinus surgery (ESS)‐assisted sphenoidal rostrum process approach surgery was performed to remove the tumor (c shows the endoscopic image after sphenoid sinus septum resection [file HED-41-440-s001.zip › hed25435-sup-0005-FigureS5h.tif]

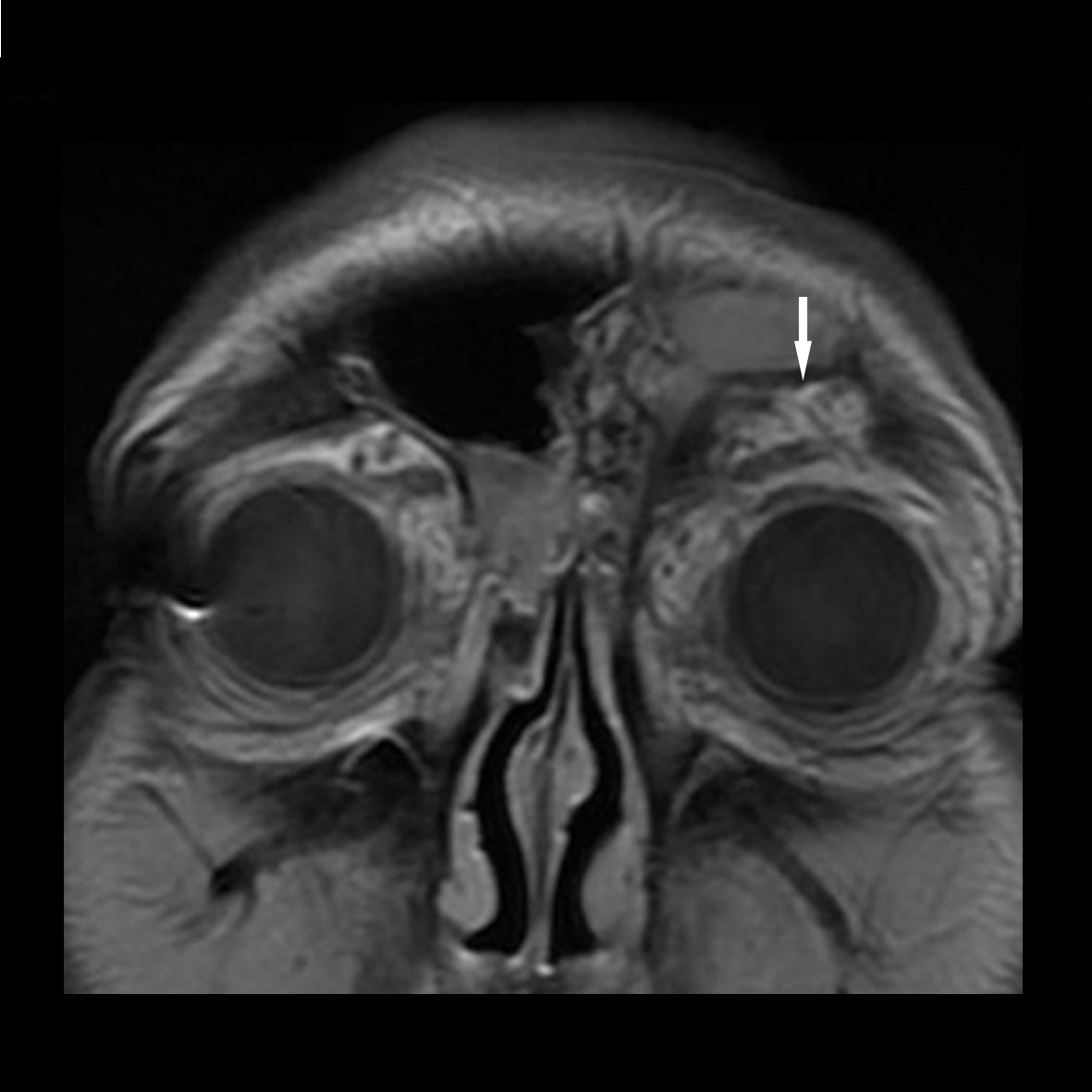

Supplement: Supplementary file 1 — Figure S1 Coronal contrast‐enhanced T1‐weighted MRI image (a) and nasal endoscopic examination (b) of a 45 years old woman, showing sinonasal inverted papilloma originating from left inferior turbinate (a, black circle). Endoscopic sinus surgery (ESS) was performed to remove the tumor, and 6 years after left inferior turbinate resection, there is no recurrence to date, as indicated by absence of tumor by CT scan (c) and endoscopy (d). Figure S2 Axial CT scan (a) and contrast‐enhanced T1‐weighted MRI image (b) of a 55 years old male, showing sinonasal inverted papilloma originating from posterior wall of left maxillary sinus (a, black circle; b, white circle). Endoscopic sinus surgery (ESS) was performed to remove the originating site of the tumor under 70° endoscopy (c ‐ tumor indicated by black arrow; d ‐ the tumor origin site after burning); with no recurrence to date 3 years after endoscopic sinus surgery. Figure S3 Axial (a) and coronal (b) contrast‐enhanced T1‐weighted MRI image of a 49 years old male, showing sinonasal inverted papilloma originating from anterior wall of left maxillary sinus (white circles). Endoscopic sinus surgery (ESS)‐assisted prelacrimal duct approach surgery was performed to remove the tumor (c ‐ black arrow indicates the originating site of the tumor, the white dotted lines indicated the lacrimal duct). Suppl. Figure 3d shows the origin site under 70° endoscopy after tumor resection (white dotted circle), A, I, M represented anterior, inferior, and medial wall of the maxillary sinus respectively; with no tumor recurrence after 5 years' follow‐up. Figure S4 Coronal CT scan (a) and contrast‐enhanced T1‐weighted MRI image (b) of a 59 years old female, showing sinonasal inverted papilloma originating from sphenoid sinus septum and affecting bilateral sinus. Endoscopic sinus surgery (ESS)‐assisted sphenoidal rostrum process approach surgery was performed to remove the tumor (c shows the endoscopic image after sphenoid sinus septum resection [file HED-41-440-s001.zip › hed25435-sup-0006-FigureS6.tif]

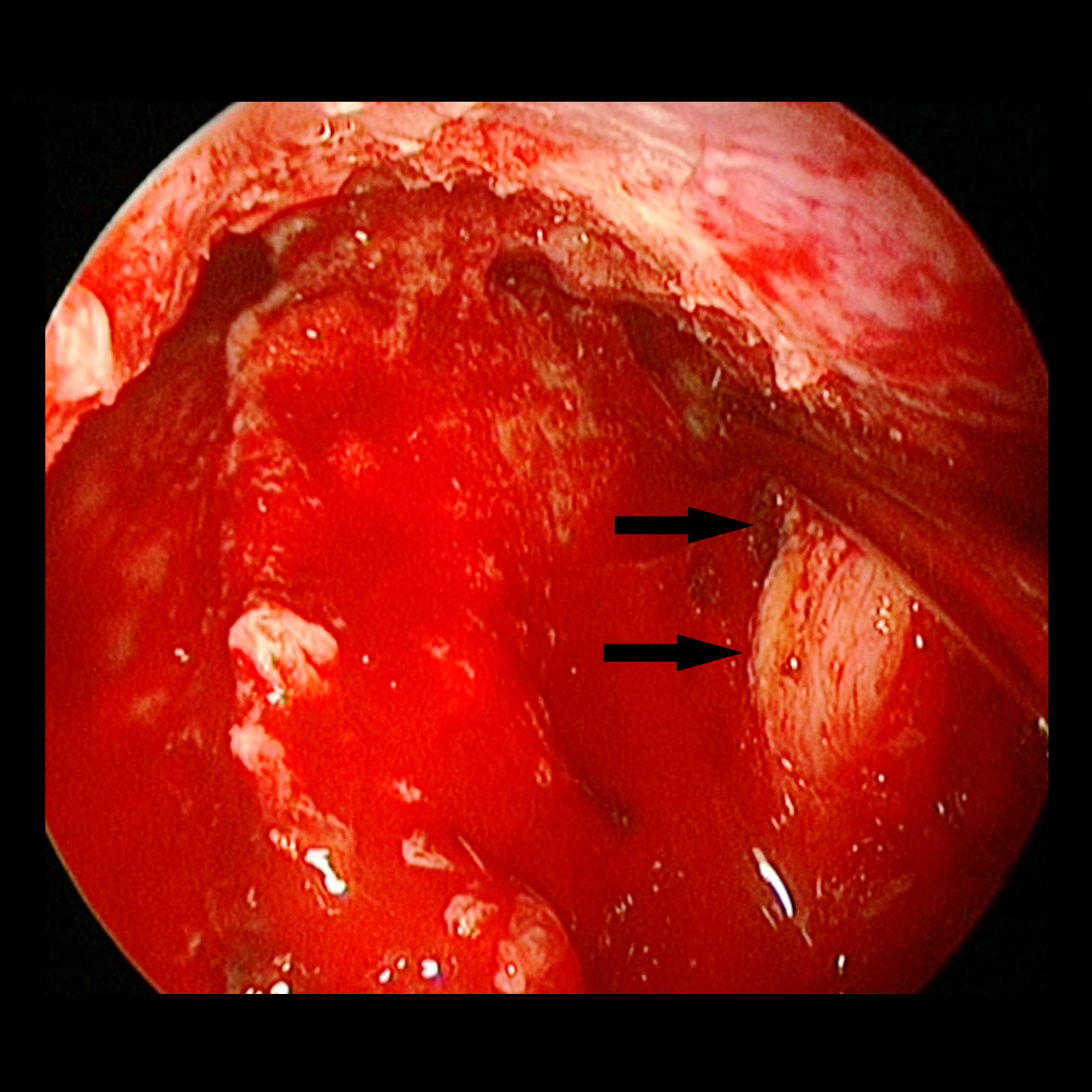

Supplement: Supplementary file 1 — Figure S1 Coronal contrast‐enhanced T1‐weighted MRI image (a) and nasal endoscopic examination (b) of a 45 years old woman, showing sinonasal inverted papilloma originating from left inferior turbinate (a, black circle). Endoscopic sinus surgery (ESS) was performed to remove the tumor, and 6 years after left inferior turbinate resection, there is no recurrence to date, as indicated by absence of tumor by CT scan (c) and endoscopy (d). Figure S2 Axial CT scan (a) and contrast‐enhanced T1‐weighted MRI image (b) of a 55 years old male, showing sinonasal inverted papilloma originating from posterior wall of left maxillary sinus (a, black circle; b, white circle). Endoscopic sinus surgery (ESS) was performed to remove the originating site of the tumor under 70° endoscopy (c ‐ tumor indicated by black arrow; d ‐ the tumor origin site after burning); with no recurrence to date 3 years after endoscopic sinus surgery. Figure S3 Axial (a) and coronal (b) contrast‐enhanced T1‐weighted MRI image of a 49 years old male, showing sinonasal inverted papilloma originating from anterior wall of left maxillary sinus (white circles). Endoscopic sinus surgery (ESS)‐assisted prelacrimal duct approach surgery was performed to remove the tumor (c ‐ black arrow indicates the originating site of the tumor, the white dotted lines indicated the lacrimal duct). Suppl. Figure 3d shows the origin site under 70° endoscopy after tumor resection (white dotted circle), A, I, M represented anterior, inferior, and medial wall of the maxillary sinus respectively; with no tumor recurrence after 5 years' follow‐up. Figure S4 Coronal CT scan (a) and contrast‐enhanced T1‐weighted MRI image (b) of a 59 years old female, showing sinonasal inverted papilloma originating from sphenoid sinus septum and affecting bilateral sinus. Endoscopic sinus surgery (ESS)‐assisted sphenoidal rostrum process approach surgery was performed to remove the tumor (c shows the endoscopic image after sphenoid sinus septum resection [file HED-41-440-s001.zip › hed25435-sup-0006-FigureS6a.tif]

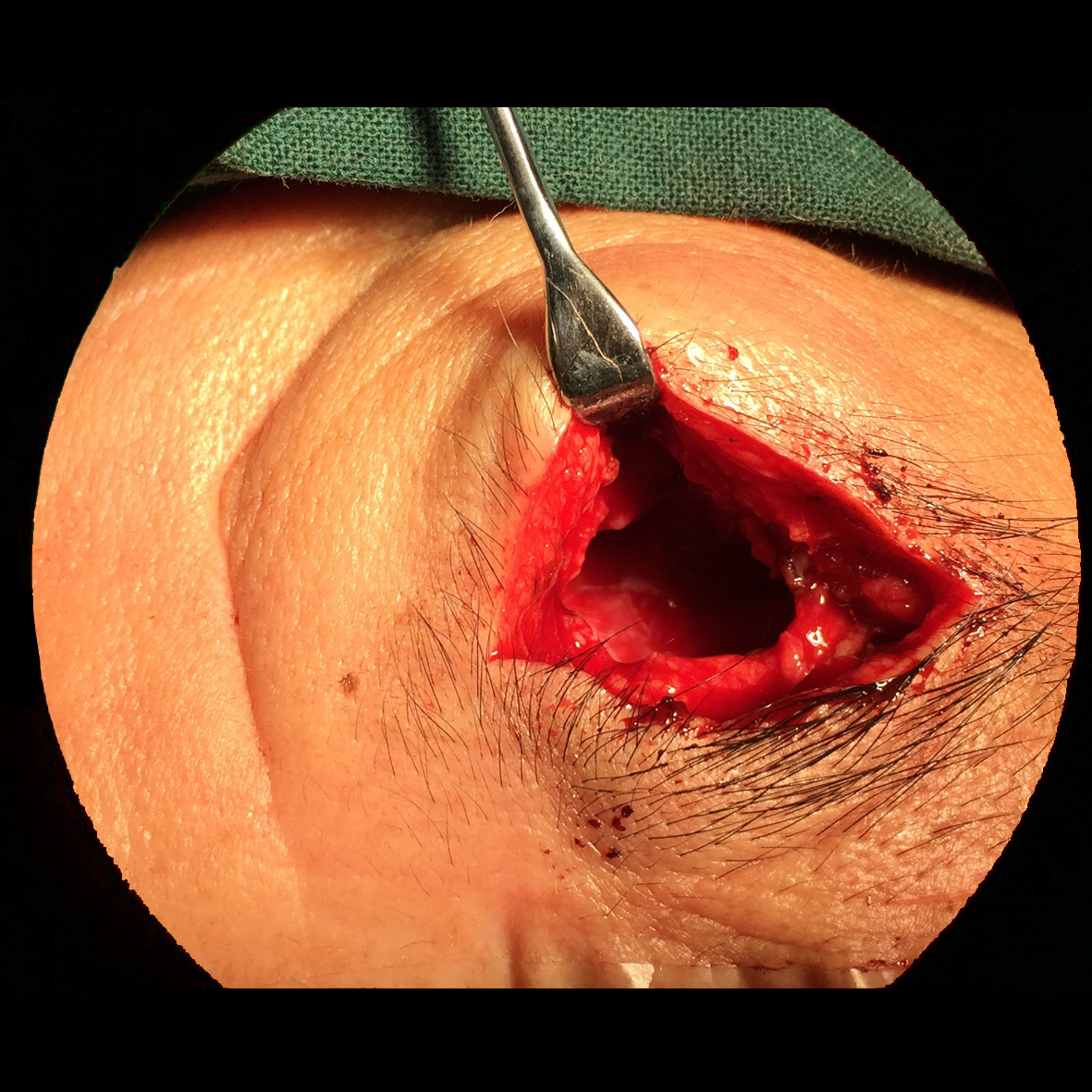

Supplement: Supplementary file 1 — Figure S1 Coronal contrast‐enhanced T1‐weighted MRI image (a) and nasal endoscopic examination (b) of a 45 years old woman, showing sinonasal inverted papilloma originating from left inferior turbinate (a, black circle). Endoscopic sinus surgery (ESS) was performed to remove the tumor, and 6 years after left inferior turbinate resection, there is no recurrence to date, as indicated by absence of tumor by CT scan (c) and endoscopy (d). Figure S2 Axial CT scan (a) and contrast‐enhanced T1‐weighted MRI image (b) of a 55 years old male, showing sinonasal inverted papilloma originating from posterior wall of left maxillary sinus (a, black circle; b, white circle). Endoscopic sinus surgery (ESS) was performed to remove the originating site of the tumor under 70° endoscopy (c ‐ tumor indicated by black arrow; d ‐ the tumor origin site after burning); with no recurrence to date 3 years after endoscopic sinus surgery. Figure S3 Axial (a) and coronal (b) contrast‐enhanced T1‐weighted MRI image of a 49 years old male, showing sinonasal inverted papilloma originating from anterior wall of left maxillary sinus (white circles). Endoscopic sinus surgery (ESS)‐assisted prelacrimal duct approach surgery was performed to remove the tumor (c ‐ black arrow indicates the originating site of the tumor, the white dotted lines indicated the lacrimal duct). Suppl. Figure 3d shows the origin site under 70° endoscopy after tumor resection (white dotted circle), A, I, M represented anterior, inferior, and medial wall of the maxillary sinus respectively; with no tumor recurrence after 5 years' follow‐up. Figure S4 Coronal CT scan (a) and contrast‐enhanced T1‐weighted MRI image (b) of a 59 years old female, showing sinonasal inverted papilloma originating from sphenoid sinus septum and affecting bilateral sinus. Endoscopic sinus surgery (ESS)‐assisted sphenoidal rostrum process approach surgery was performed to remove the tumor (c shows the endoscopic image after sphenoid sinus septum resection [file HED-41-440-s001.zip › hed25435-sup-0006-FigureS6b.tif]

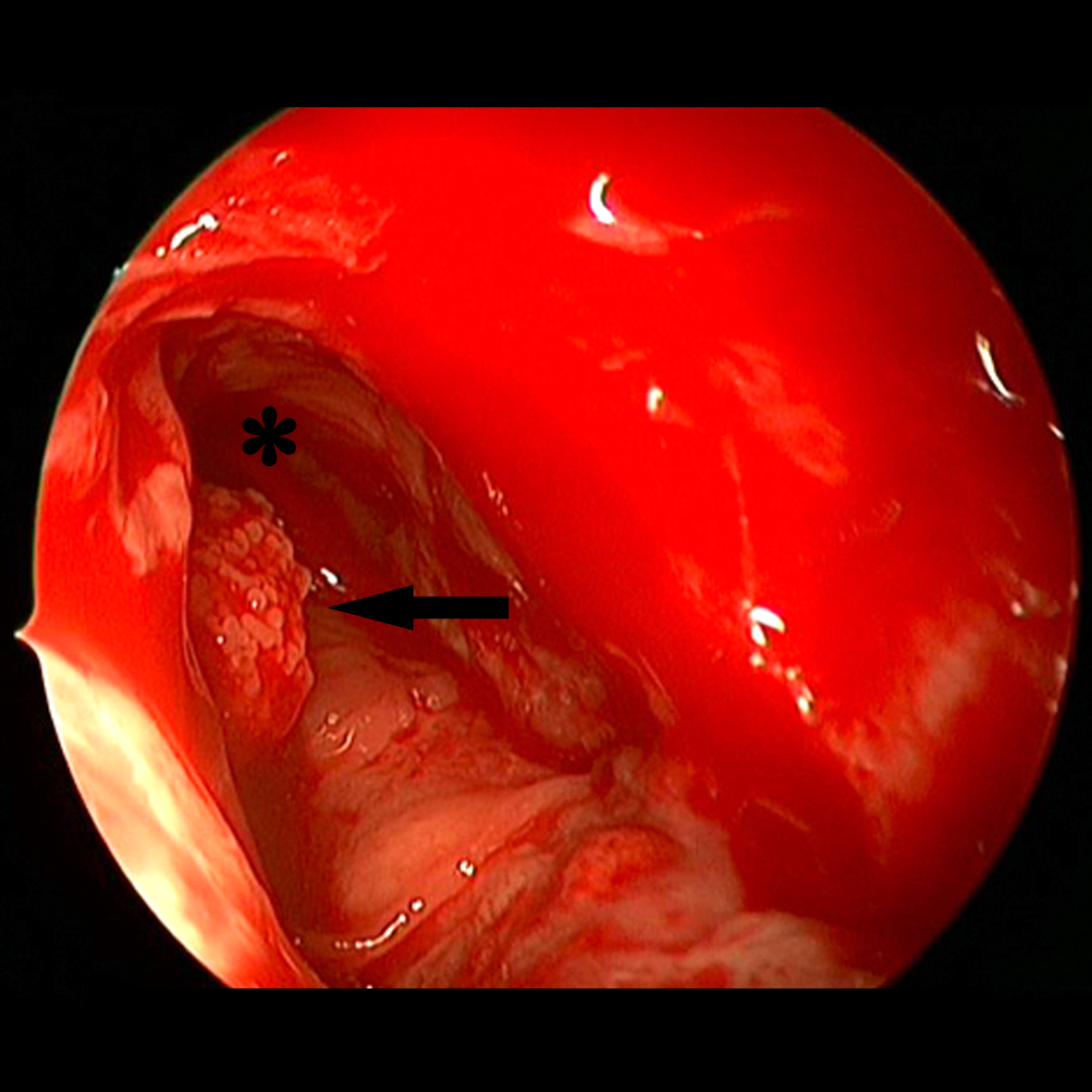

Supplement: Supplementary file 1 — Figure S1 Coronal contrast‐enhanced T1‐weighted MRI image (a) and nasal endoscopic examination (b) of a 45 years old woman, showing sinonasal inverted papilloma originating from left inferior turbinate (a, black circle). Endoscopic sinus surgery (ESS) was performed to remove the tumor, and 6 years after left inferior turbinate resection, there is no recurrence to date, as indicated by absence of tumor by CT scan (c) and endoscopy (d). Figure S2 Axial CT scan (a) and contrast‐enhanced T1‐weighted MRI image (b) of a 55 years old male, showing sinonasal inverted papilloma originating from posterior wall of left maxillary sinus (a, black circle; b, white circle). Endoscopic sinus surgery (ESS) was performed to remove the originating site of the tumor under 70° endoscopy (c ‐ tumor indicated by black arrow; d ‐ the tumor origin site after burning); with no recurrence to date 3 years after endoscopic sinus surgery. Figure S3 Axial (a) and coronal (b) contrast‐enhanced T1‐weighted MRI image of a 49 years old male, showing sinonasal inverted papilloma originating from anterior wall of left maxillary sinus (white circles). Endoscopic sinus surgery (ESS)‐assisted prelacrimal duct approach surgery was performed to remove the tumor (c ‐ black arrow indicates the originating site of the tumor, the white dotted lines indicated the lacrimal duct). Suppl. Figure 3d shows the origin site under 70° endoscopy after tumor resection (white dotted circle), A, I, M represented anterior, inferior, and medial wall of the maxillary sinus respectively; with no tumor recurrence after 5 years' follow‐up. Figure S4 Coronal CT scan (a) and contrast‐enhanced T1‐weighted MRI image (b) of a 59 years old female, showing sinonasal inverted papilloma originating from sphenoid sinus septum and affecting bilateral sinus. Endoscopic sinus surgery (ESS)‐assisted sphenoidal rostrum process approach surgery was performed to remove the tumor (c shows the endoscopic image after sphenoid sinus septum resection [file HED-41-440-s001.zip › hed25435-sup-0006-FigureS6c.tif]
